# Dexmedetomidine Mitigates Sevoflurane-Induced Neurodevelopmental Effects in Paediatric Anaesthesia: A Meta-Analysis and Preclinical Study

**DOI:** 10.1007/s11481-025-10273-8

**Published:** 2026-01-07

**Authors:** Hsuan-Chih Lao, Chia-Wei Huang, Ssu-Han Wang, Yen-Lin Su, Chien-Hui Chang, Cheng-Yen Liao, Jen-Chieh Wu, Ying-Chun Lin, Jin-Wu Tsai

**Affiliations:** 1 Institute of Brain Science, College of Medicine, National Yang Ming Chiao Tung University, No.155, Sec.2, Linong Street, Taipei, 112 Taiwan; 2 Department of Anesthesiology, Mackay Memorial Hospital, Taipei, Taiwan; 3 Department of Medicine, MacKay Medical University, New Taipei, Taiwan; 4 Advanced Therapeutics Research Center, National Yang Ming Chiao Tung University, Taipei, Taiwan; 5 Institute of Clinical Medicine, College of Medicine, National Yang Ming Chiao Tung University, Taipei, Taiwan; 6 Department of Medical Education, Taipei Veterans General Hospital, Taipei, Taiwan; 7 School of Medicine, College of Medicine, National Yang Ming Chiao Tung University, Taipei, Taiwan; 8 Department of Medical Research, Mackay Memorial Hospital, Taipei, Taiwan; 9 Institute of Epidemiology and Preventive Medicine, College of Public Health, National Taiwan University, Taipei, Taiwan; 10 Mackay Medicine, Nursing and Management College, Taipei, Taiwan; 11 Brain Research Center, National Yang Ming Chiao Tung University, Taipei, Taiwan; 12 Department of Biological Science and Technology, College of Biological Science and Technology, National Yang Ming Chiao Tung University, Hsinchu, Taiwan

**Keywords:** Sevoflurane, Emergence agitation, Preschooler, Dexmedetomidine, Dendritic spine, Neuronal migration

## Abstract

Sevoflurane (Sevo) anaesthesia in children is linked to an increased incidence of postoperative emergence agitation (EA) and potential neurotoxicity in developing brains. However, the specific risks of subanaesthetic foetal or neonatal exposure to Sevo remain unclear. This study evaluates the safety and efficacy of combining dexmedetomidine (Dex) with Sevo to manage EA in paediatric anaesthesia. A systematic review and meta-analysis of randomized controlled clinical trials involving children under 8 years old revealed that Dex significantly reduces EA incidence when administered via intravenous, perineural, and intranasal routes. Using *in utero* electroporation, we found that pregnant mice exposed to 2.5% Sevo at embryonic days 14.5 and 15.5 exhibited transient neuronal migration deficits, with 25% of neurons delayed in deeper cortical layers. However, these neurons migrated to the cortex by postnatal day 8. Neonatal mice exposed to 2.5% Sevo experienced a 10% reduction in dendritic spine density in adolescence, associated with impaired somatosensory function, as assessed by the Von Frey test. Remarkably, Dex pretreatment ameliorated these pathological and functional changes. Thus, foetal or neonatal Sevo exposure can delay neuronal migration and reduce dendritic spine density. Dex co-administration effectively mitigates these adverse outcomes, supporting its potential use in paediatric anaesthesia to protect developing brains.

**Graphical abstract:**

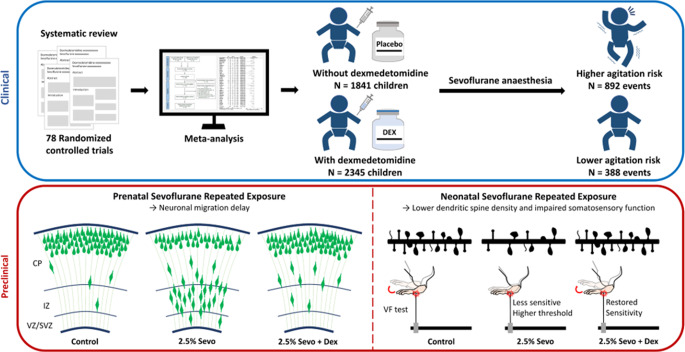

**Supplementary Information:**

The online version contains supplementary material available at 10.1007/s11481-025-10273-8.

## Introduction

The clinical need for early exposure to anaesthetics is inevitable (Shi et al. 2018), particularly in cases of paediatric and foetal surgery, such as inguinal hernia repair (Lao et al. 2012; Chang et al. 2016) and fetoscopic laser coagulation of placental anastomoses in twin-to-twin transfusion syndrome (Bergh et al. 2021). Long-term observations of infants and young children who underwent foetal or paediatric general anaesthesia suggest that repeated exposure to anaesthetics is associated with developmental delays in gross motor function, as well as deficits in processing speed and motor coordination (Zaccariello et al. 2019; Kobayashi et al. 2020). Most recently, a study in preschool-aged children reported that multiple exposures to general anaesthesia were correlated with lower general intelligence scores and poorer performance across several neurodevelopmental domains (Xin et al. 2025). Emergence agitation (EA), characterized by a dissociated state of attention and awareness of surroundings, along with disorientation, restlessness, excitation, non-purposeful movement, inconsolability, thrashing, and incoherence during early recovery from general anaesthesia (McCann and Soriano 2019; Lee and Sung 2020), raises concerns about possible changes in cortical integration caused by inhalational anaesthetics, especially in preschoolers (Bonhomme et al. 2012). Although EA is generally temporary, it is extremely distressing for children, parents, and health professionals.

Sevoflurane (Sevo) is one of the most commonly used anaesthetics in foetal and paediatric surgeries due to its mild odour, minimal irritation, and superior bronchodilatory effect on smaller airways. However, the incidence of agitation during recovery from Sevo-based anaesthesia is generally much higher compared to other anaesthetic agents such as halothane (Kuratani and Oi 2008) and propofol (Kanaya et al. 2014). Although the exact cause of frequent EA after Sevo is unclear, possible factors include Sevo-induced seizure activity and quicker emergence time. Over the past decade, emerging preclinical evidence from studies on prenatal and neonatal rodent brains has indicated that single or multiple exposures to Sevo may induce neurotoxicity, leading to cell apoptosis in the hippocampus (Zheng et al. 2013; Wang et al. 2022; Jiang et al. 2023) and cortex (Song et al. 2017; Chen et al. 2021; Areias et al. 2023). Despite the warnings issued by the US Food and Drug Administration (FDA) in 2016 regarding anaesthesia-induced neurotoxicity in developing brains (FDA, 2016; Jevtovic-Todorovic 2017), Sevo continue to be widely used due to its advantages in pharmacodynamics and pharmacokinetics. However, little is known about the early signs of neuronal damage in the cortex, such as the reduction of dendritic spines, which is associated with seizure (Jean et al. 2023) and psychomotor agitation (Kim et al. 2015).

Dendritic spines are small protrusions on the surface of dendrites and constitute the postsynaptic structure in various neurones, including cortical pyramidal neurones (Chidambaram et al. 2019). Proper dynamics and maturation of dendritic spines during development are critical for establishing functional connectivity in the cortex (Hasegawa et al. 2015). In the developing brain, dendritic spines undergo a series of developmental processes from childhood to adulthood, including spinogenesis, pruning, and maintenance (Kasai et al. 2010). During the spinogenesis process, the rate of spine formation exceeds that of elimination, resulting in a growing number of spines that can establish synaptic connections in response to learning and memory events, as well as multiple sensory stimulations from the extrinsic environment (Ma et al. 2016). These dynamic modifications of dendritic spines are tightly regulated by neurones, astrocytes, and microglia (Stein and Zito 2019). Disruption of this regulatory mechanism, due to neuroinflammation, glial modulation, and abnormal intracellular signals, may result in decreased spine density owing to an enhanced spine elimination rate. Previously, several studies have reported that repeated neonatal exposure to Sevo in neonatal mice may lead to a prolonged reduction in dendritic spine density of hippocampal neurones in adult mice (Ji et al. 2015) and rats (Amrock et al. 2015; Jia et al. 2016; Ju et al. 2016). However, the long-term effects of repeated Sevo anaesthesia after birth on dendritic spine development in the cortex remain unclear.

In addition to the observed neuronal death and postsynaptic deficits in both foetal and adolescent mice, the impacts of maternal exposure to Sevo on cortical development, such as neurogenesis and neuronal migration, have been reported in several studies (Chai et al. 2019, 2020; Jiang et al. 2021). Jiang et al. analysed pregnant mice at E14.5, subjected to a single Sevo exposure for 6 h, and found that the interkinetic nuclear migration (INM) process of neural progenitor cells (i.e., radial glial cells) was impaired, leading to abnormal cell cycle progression during early development (Jiang et al. 2021). In studies focussing on the postmitotic stages, Chai et al. reported that repeated Sevo treatments in pregnant mice at E14.5 and E15.5 for 2 h per day led to a delay in neuronal migration at E18.5 (Chai et al. 2019). Since neuronal migration is a crucial developmental process in the formation of the cerebral cortex, any disruption or delay in this process may lead to various cortical malformations (Ross and Walsh 2001; Romero et al. 2018). However, the subcellular mechanisms of these effects of Sevo exposure remain elusive (Chai et al. 2019, 2020).

Given the high incidence of EA, researchers have focused on providing effective prophylactic treatments, including propofol (Chen et al. 2013; Messieha 2013), midazolam (Breschan, 2007; Kim et al. 2011), ketamine (Chen et al. 2013), nonsteroidal anti-inflammatory drugs (NSAIDs) (Kim et al. 2013), fentanyl (Demirbilek et al. 2004), nitrous oxide (N_2_O) (Shibata et al. 2005), magnesium (Abdulatif et al. 2013), regional block (Sinha and Sood 2012), melatonin (Ozcengiz et al. 2011), and α2 adrenergic receptor agonist(Ghosh et al. 2011). Dexmedetomidine (Dex), an α2 adrenergic receptor agonist, is now commonly used for sedation in paediatric patients. Beyond its sedative properties, Dex has demonstrated potential neuroprotective effects against neurotoxicity and neurodegeneration induced by non-volatile or volatile general anaesthetics (Andropoulos 2023; Tsivitis et al. 2023). These include the attenuation of perioperative neurocognitive disturbances, such as agitation and delirium, along with reductions in excitotoxic neuronal injury, apoptotic cell death following cerebral ischemia, and suppression of neuroinflammatory responses triggered by general anaesthetic exposure (Engelhard et al. 2003; Dahmani et al. 2008; Sanders et al. 2009). Such actions may contribute mechanistically to the prevention of EA and provide a conceptual framework linking EA to longer-term neurodevelopmental consequences. Supporting this notion, recent experimental work showed that Dex exhibited superior safety and neuroprotective effects against Sevo-induced neurotoxicity in neonatal rats (Lee et al. 2017a). These findings suggest that Dex pretreatment may also mitigate the early signs of neuronal death, such as morphological changes in synaptic structures.

In this study, we systematically reviewed the literature on the effects and safety of combining Sevo and Dex during anaesthesia in children, particularly those under 8 years old. Furthermore, we performed a meta-analysis of randomised controlled trials to evaluate the beneficial effects of Dex on the quality of postoperative recovery. Despite these clinical insights, there remains a scarcity of data regarding the long-term neuroprotective effects of Dex in the context of Sevo exposure during early neurodevelopment. To address this gap, we investigated the long-term effects of repeated foetal and neonatal exposure to Sevo on dendritic morphology and neuronal function in the developing cortex. To explore potential clinical applications, we provided in vivo experimental evidence supporting the benefits of using Dex as a pretreatment before anaesthesia. This intervention offers a potential pathway for mitigating brain damage resulting from anaesthesia during foetal and neonatal surgeries.

## Materials and Methods

### Article Retrieval

The systematic review was conducted following the Preferred Reporting Items for Systematic Reviews and Meta-Analyses (PRISMA) guidelines (Page et al. 2021). A comprehensive search for randomized controlled clinical trials (RCTs) involving preschool children was performed in biomedical databases including MEDLINE, Cochrane Library, Embase up to September 12, 2023. The search terms used included: (“dexmedetomidine” or “Precedex”), (“emergence agitation” or “emergence delirium” or “emergence excitement”), (“child” or “infant” or “paediatric”), along with their Mesh terms, Emtree terms, and synonyms. The primary citations were filtered to include only RCTs involving humans, with no restriction on publication year and limited to English-language publications. References of related review articles and pairwise meta-analyses were manually searched to identify additional eligible studies. The detailed search strategy for each database is provided in supplementary information (Supplementary Table S1).

### Inclusion and Exclusion Criteria of Articles

References selected from the initial search were screened for eligibility using the following criteria: [1] subjects were children undergoing general anaesthesia with Sevo; [2] subjects were children below eight years old (median ± standard deviation or median ± interquartile range); [3] the study provided a comparison between Dex as an intervention and a placebo and/or active comparators; [4] the study was designed as an RCT. The exclusion criteria were: [1] no validated method for evaluating EA was mentioned; [2] the data was questionable or inconsistent; [3] conference abstracts and other summary publications; [4] duplicated articles or data (Supplementary Table S2).

### Data Extraction

The eligibility, methodologies, and publication data of the included trials were independently assessed by two co-authors (S.-H. Wang and H.-C. Lao). Discrepancies were resolved through discussion. Full texts were examined for trial that appeared qualified. The primary outcome was the incidence of EA after the administration of Sevo-based anaesthesia and various prophylactic interventions. The diagnosis of EA was based on criteria from individual studies, including the Pediatric Anesthesia Emergence Delirium scale (PAED) (Sikich and Lerman 2004), Watcha scale (Watcha et al. 1992), Aono scale (Aono et al. 1997), Cole scale (Cole et al. 2002), Cravero scale (Locatelli et al. 2013), Ramsay sedation score (Zhang and Li 2018), 5-point scale_Peng 2015 (Peng et al. 2015), 5-point scale_Scheinin 1989 (Scheinin et al. 1989), 3-point scale_Abdel-Ghaffar 2018 (Abdel-Ghaffar et al. 2018), and Restlessness score (Liang et al. 2022). The following data were extracted: primary author, publication year, types of surgery, characteristics of the children, patient age, American Society of Anesthesiologists (ASA) physical status, anaesthesia used, premedication, intervention and control group details (type, dosage, timing, route), parental separation, pain assessment method, extubation time, emergence time, time to discharge from PACU, and several adverse events related to anaesthesia recovery, including rescue analgesia, postoperative nausea and vomiting (PONV), hypotension, and bradycardia.

### Risk-of-Bias Assessment (Meta-Analysis)

Two reviewers (S.-H. Wang and H.-C. Lao) independently evaluated the quality of the included trials using the Cochrane Risk of Bias 2.0 tool. This tool examines the following items: [1] Randomization process, [2] Intended intervention, [3] Missing outcome data, [4] Measurement of the outcome, [5] Selection of the reported result. Each item was judged for low, unclear, or high risk of material bias. In case of discrepancies, a third author (Y.-C. Lin) was involved to reach a consensus.

### Statistical Analysis

All meta-analyses were performed using Review Manager (version 5.4.1, Cochrane, Collaboration, Copenhagen, Denmark). Pooled effects were presented as relative risk (RR) for dichotomous variables and mean difference (MD) for continuous variables. Statistical heterogeneity across studies was assessed using Cochran’s Q test and quantified with the I^2^ statistic. A random-effects model was if difference in study designs was evident, the p-value for Cochran’s Q test was < 0.1, or I^2^ exceeded 50%; otherwise, a fixed-effect model was used. To evaluate the robustness of the finding, sensitivity analyses were conducted based on study sample size to mitigate potential biases from small-sample studies. An additional sensitivity analysis was conducted excluding studies with a high risk of bias to validate the results. All statistical outcomes were reported with 95% confidence intervals (CI), and a two-sided p-value < 0.05 was considered statistically significant. Funnel plots were visually inspected to assess publication bias if at least ten trials were identified.

Trial sequential analysis (TSA) was performed to reduce the risks of random errors, increase the robustness of the meta-analyses, and determine whether the current sample size was sufficient (Shah and Smith 2020). The required information size (RIS) and trial sequential monitoring boundaries (TSMB) were calculated to determine whether the evidence in our meta-analysis was reliable and conclusive. If the cumulative Z-curve entered the futility area or crossed the TSMB, the anticipated intervention effect showed firm evidence; otherwise, the evidence was rated as absent. The risk of a type 1 error was set to 5% with a power of 80%. For dichotomous outcome of EA, we set the effect measure as ‘Relative Risk’ and the model as ‘Random-effects (DL)’ or ‘Fixed-effects’ in TSA software. Relative risk reduction (RRR) was defined as 30%, based on standard convention to reach adequate clinical significance (Turner et al. 2013). The incidence in the control arm was calculated from the average incidence in the control group, and heterogeneity correction was set as model variance-based. The trial sequential analysis software (version 0.9.5.10 beta) was used the analysis.

### Animals and Ethics Approval

Pregnant CD1 (ICR) mice were purchased from BioLASCO, Taipei, Taiwan (full accreditation awarded by AAALAC International) and housed in the Laboratory Animal Center of National Yang Ming Chiao Tung University (NYCU) from embryonic day (E) 11.5. Our study examined male and female animals, and similar findings are reported for both sexes. All animal studies were conducted in compliance with protocols approved by the Institutional Animal Care and Use Committee (IACUC) of NYCU (IACUC No. 1110520 and 1100426).

### ***In Utero*** Electroporation (IUE)

The procedures of IUE were conducted as previously described (Huang et al. 2023). Briefly, pregnant mice were initially anesthetized with 4% isoflurane (Iso) in an induction chamber, followed by maintenance with 1.5% Iso via a face mask. After laparotomy, the uterus was gently externalized, and each embryo was randomly injected with 0.5 µl of plasmid DNA into the lateral ventricle. For neuronal labeling, US2-GFP (0.6 µg/µl) was used to visualize migrating neurons in the somatosensory cortex of foetal brains, and pCAG-eYFP (1.5 µg/µl) was used to trace dendritic structures in postnatal mice. Electroporation was performed with the following parameters: 40 V, five 50-ms pulses, delivered at 450-ms intervals. The uterine horns were repositioned into the abdominal cavity, and the incision was sutured. Embryos were allowed to develop normally and delivered naturally. Electroporated brains were harvested at embryonic day 18.5 (E18.5), postnatal day 8 (P8), P21, and P30 for subsequent analyses.

Based on extensive experience in our laboratory, proficient IUE does not result in apparent or quantifiable damage to the developing brain. We have previously assessed cell death using Caspase-3 immunostaining (Chen et al. 2019) and evaluated gliosis using Iba1 and GFAP staining to detect microglial and astrocytic activation, respectively (Huang et al. 2023), and found no statistically significant increases. A small proportion of embryos (< 10% per litter) may develop hydrocephalus or undergo foetal resorption, typically attributable to surgical variability or the maternal health condition. These cases were excluded from the final analysis. The survival rate following proficient IUE exceeds 90%. Miscarriage occurs in fewer than 5% of pregnancies, and foetal resorption remains rare. Postnatal development was monitored, and no significant differences in body weight, body length, or general health were observed between IUE-treated and control pups.

### Brain Section and Immunofluorescence Staining

Detailed procedures of immunofluorescence staining in brain slices have been described previously (Tsai et al. 2020a, b; Huang et al. 2023). The sources of primary antibodies used in this study were: anti-NeuN rabbit polyclonal antibody (1:500, Millipore, ABN78), anti-BRN2 mouse monoclonal antibody (1:500, Santa Cruz Biotechnology, sc-393324), fluorescent-dye conjugated secondary antibodies AlexaFluor™ 546 (1:500, Thermo Fisher Scientific, A-11010). Nucleus staining dye: DAPI (4’,6-diamidino-2-phenylindole, dihydrochloride, 1:1000, Thermo Fisher Scientific, 62247). Mounting buffer: VECTASHIELD^®^ Mounting Media (Vector Laboratories, H-1000).

### General Anaesthesia

A sevoflurane (Sevo) concentration of 2.5% was selected for this study based on previous research investigating Sevo-induced neurodevelopmental effects in neonatal rodent models (Jiang et al. 2017; Wu et al. 2018; Zhang et al. 2020). This dosage aligns with clinical practice, as the minimum alveolar concentration (MAC) of Sevoflurane in children aged 3–5 years is approximately 2.5%–3% (Katoh and Ikeda 1992). For the gestational exposure protocol, pregnant ICR mice at embryonic day 14.5 (E14.5) were first anesthetized with 1.5% isoflurane and underwent IUE, which typically lasted approximately 30 min. Following a brief recovery period (~ 10 min), the mice were subjected to 1 h of general anaesthesia with 2.5% Sevo delivered in 100% oxygen. The same exposure was repeated the following day (E15.5). This dual-exposure model was designed to mimic repeated anaesthetic interventions during critical periods of foetal cortical development—a scenario occasionally encountered in clinical practice. Our approach is comparable with established protocols in prior studies employing both rodent and large animal models, including mouse (Chung et al. 2017a; Fang et al. 2017; Lee et al. 2017b; Chai et al. 2019; Wei et al. 2024), rat (Zhang et al. 2020; Jiang et al. 2023), rabbit (Van der Veeken et al. 2019), and ovine (Van der Veeken et al. 2019).

For the protocol of repeated neonatal Sevo exposure, E14.5 pregnant ICR mice underwent IUE as mentioned above. Their offspring was exposed to 2-hr general anaesthesia (1% or 2.5% Sevo with 60% pure oxygen) on postnatal days (P) 6, 9, and 12. While the use of 60% oxygen was initially tested for both prenatal and neonatal exposures to reduce the potential for oxygen toxicity, we observed a markedly increased mortality rate (~ 50%) among pregnant mice subjected to Sevo exposure with 60% oxygen following IUE. Therefore, to maintain animal welfare and ensure experimental reproducibility, we opted for 100% oxygen in all prenatal procedures—a practice consistent with standard IUE protocols and previously published studies (Chung et al. 2017a; Fang et al. 2017; Chai et al. 2019; Bleeser et al. 2023).

### Dexmedetomidine Pretreatment

To assess the neuroprotective effects of Dex, pregnant ICR mice received intraperitoneal (IP) injections of Dex at a dose of 5 µg/kg, administered 10 min prior to each session of Sevoflurane anaesthesia on E14.5 and 15.5. The same dosage and administration route were applied to neonatal mice, with Dex administered prior to each anaesthetic exposure on P6, P9, and P12. This dosing regimen was based on previous studies demonstrating that Dex doses exceeding 5 µg/kg under 2.5% Sevo exposure are associated with increased neonatal mortality over time, whereas doses of 1–5 µg/kg Dex confer neuroprotection without adverse effects during shorter (2-hour) exposures to 2.5% Sevo (Perez-Zoghbi et al. 2017). Accordingly, a dose of 5 µg/kg was chosen to provide an optimal balance between efficacy and safety in both prenatal and neonatal models.

### Von Frey Test

The Von Frey test used in this study was modified from the protocol previously described (Takatsuru et al. 2015). Mice first underwent a 30-mins habituation period in the plexiglass chamber with a wire mesh floor daily for 5 days prior to the test. After this habituation, a mechanical stimulus was delivered perpendicularly to the plantar surface of the hind paws through a 0.5 mm steel rod until the mice withdrew their paws. The force of the stimulus was gradually increased from 0 to 15 µg at a rate of 5 g/s, and the nociceptive threshold of these mice was automatically measured by a Dynamic Plantar Aesthesiometer (Ugo Basile, Italy). The threshold for each mouse was obtained from the average value of three consecutive trials.

### Dendritic Spine Analysis

Mouse brains electroporated with US2-GFP and pCAG-eYFP constructs were fixed, sectioned, and mounted. Approximately ten to fifteen basal dendrites of cortical layer II/III pyramidal neurons in the somatosensory cortex were randomly imaged from eight brain slices of each mouse by confocal microscopy (LSM700, Carl Zeiss). The dendrites were analysed utilising semi-automated and artificial intelligence (AI)-assisted algorithms (Huang et al. 2023). Spine density results in each group consist of thousands of dendritic spines from more than three mice. Image parameters: frame size: 1024 (X)*1024 (Y); pixel size: 0.078 μm*0.078 μm*0.42 μm (X, Y, Z).

## Results

### Effectiveness of Dex in Reducing EA Following Sevo Anaesthesia: A Meta-analysis

**Fig. 1:**
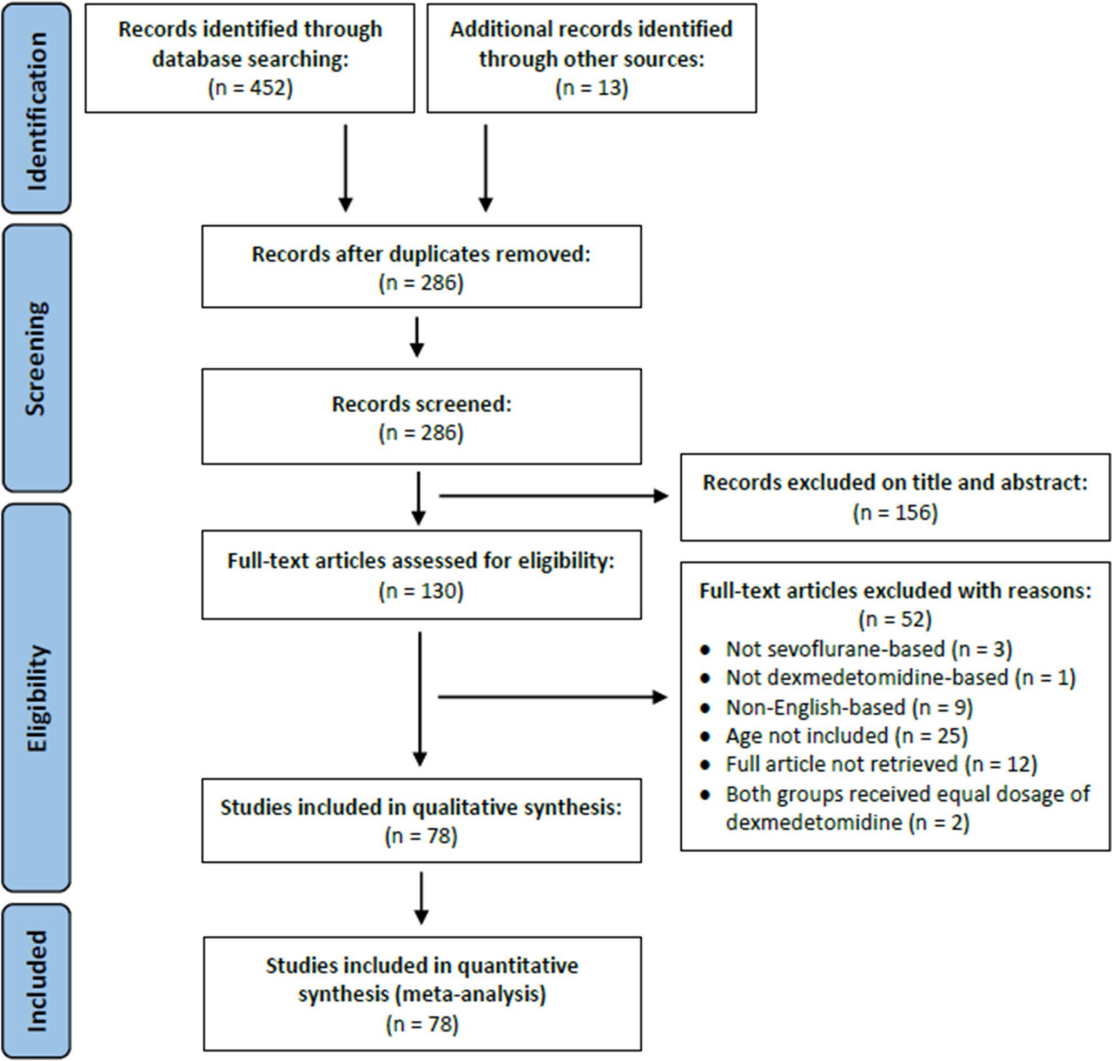
PRISMA flow chart of meta-analysis

A total of 452 related articles were retrieved in the initial search. After removing duplicates and excluding 156 citations based on titles and abstracts, 130 studies were accessed in detail. Subsequently, 52 articles were excluded for not meeting the inclusion and exclusion criteria (Supplementary Table S2). Finally, 78 full-text, independent articles were included in our meta-analysis. The literature screening strategy is illustrated in Fig. 1, while the detailed characteristics of the included 78 RCTs are presented in supplementary information (Supplementary Table S3). The effectiveness of Dex compared to placebo was evaluated using pooled data from 4186 children, including 1280 events of EA. Dex was administered through various routes, including intravenous route (bolus and continuous infusion), perineural, intranasal, and oral.

**Fig. 2:**
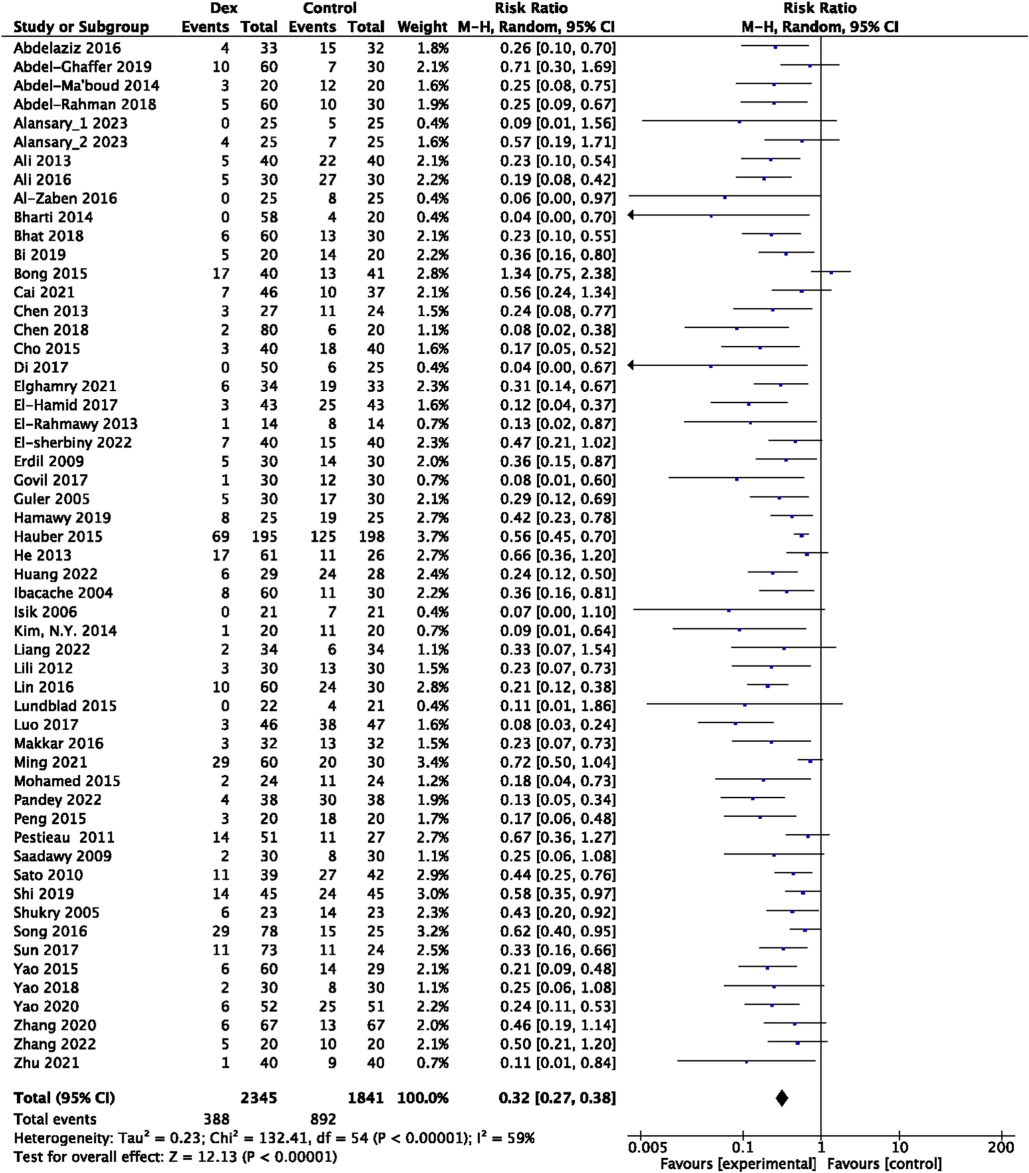
Risk of EA in children with or without Dex following Sevo exposure. Forest plot for EA incidence: Dex vs. control (Placebo)

**Fig. 3:**
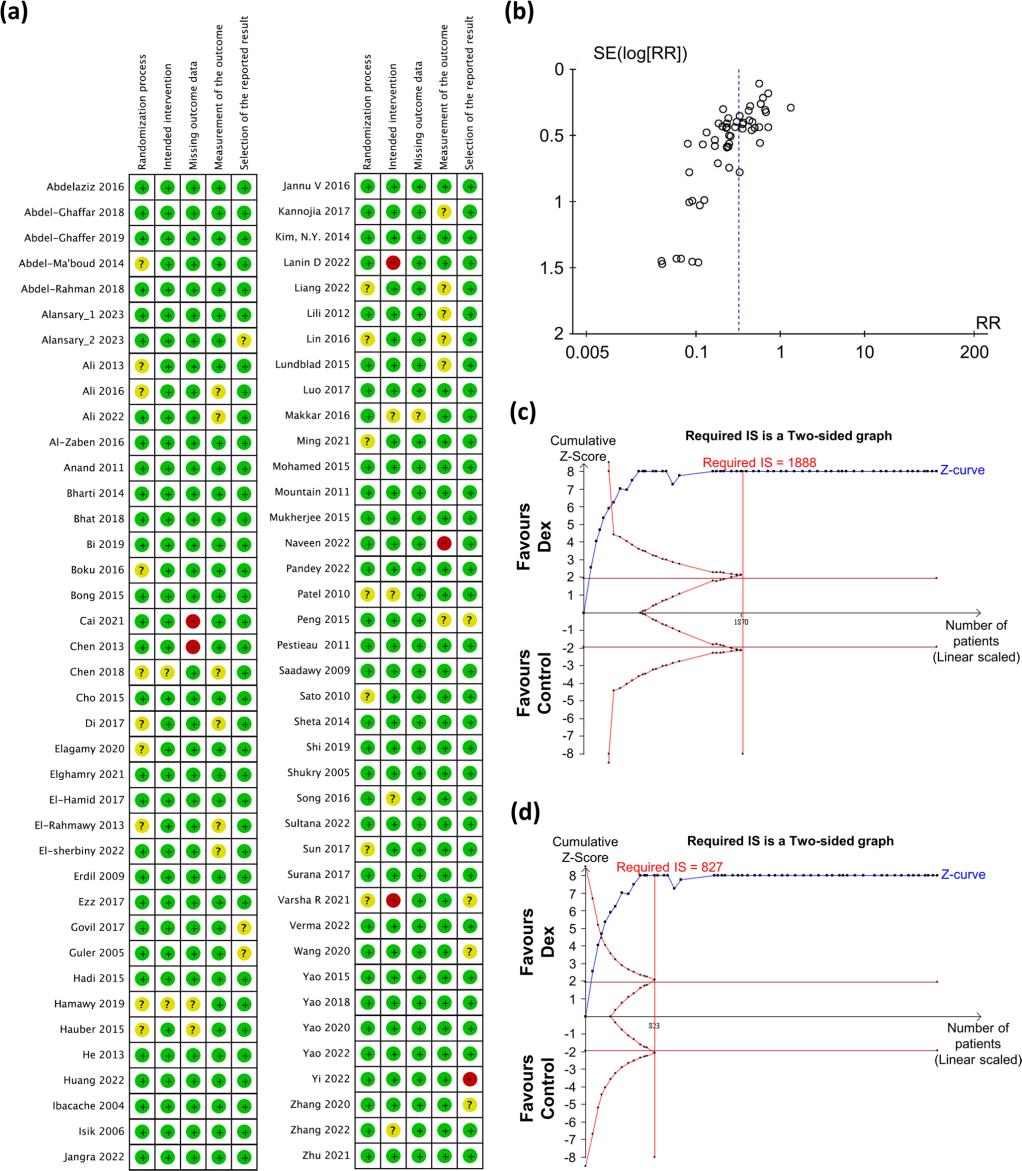
Risk of bias in each included randomised controlled clinical trial. (**a**) Summary of risk of bias assessment. (**b**) Funnel plot for publication bias test of EA incidence. (**c**) (**d**) Trial sequential analysis for EA incidence: Dex vs. control (Placebo). SE (Log [RR]), standard error (Log [risk ratio]). RR, risk ratio. Required IS, required information size

Among these 78 RCTs, 55 studies involving 4,186 patients, including both placebo and Dex groups, were selected for assessing the effects of Dex compared to placebo in reducing the risk of EA in preschool children (under eight years old) (Fig. 2). Remarkably, Dex was associated with a decreased incidence of Sevo-related EA, with a pooled RR of 0.32 (95% CI: 0.27–0.38, *P* < 0.00001) and moderate heterogeneity as indicated by Cochran’s Q test (*P* < 0.00001, I^2^ = 59%,) (Fig. 2). Sensitivity analyses based on study sample size yielded consistent results, with pooled RRs of 0.25 (95% CI: 0.20–0.32; *P* < 0.00001) and 0.35 (95% CI: 0.28–0.44; *P* < 0.00001), respectively (Supplementary Table S6; Supplementary Fig. S1). Exclusion of high-risk-of-bias studies resulted in comparable results, further supporting the reliability of the pooled estimate (Supplementary Fig. S2). The risk of bias for each RCT was assessed, revealing that most studies were ranked as having a comparably low risk of bias (Fig. 3a). A funnel plot was used and checked visually, indicating the reliability of the result (Fig. 3b). To further strengthen the evidence, TSA was performed under a conservative threshold of 20% anticipated RRR. The RIS of 1,888 was reached, with the cumulative Z-curve crossing both the conventional boundary and TSMB (Fig. 3c). TSA confirmed that both the conventional boundary and TSMB were crossed, with the RIS (calculated as 827) being reached by the cumulative Z-curve (Fig. 3d; Supplementary Table S4). TSA provided firm evidence that Dex is superior to placebo in reducing the incidence of EA in preschool children undergoing Sevo-based anaesthesia.

To explore the impact of known confounding factors on the efficacy of Dex in preventing Sevo-related EA, we also performed subgroup analyses based on different administration routes (Supplementary Fig. S3a). The pooled results indicated that intravenous (RR: 0.31, 95% CI: 0.25–0.40, *P* < 0.00001, I^2^ = 62%), perineural (RR: 0.22, 95% CI: 0.14–0.33, *P* < 0.00001, I^2^ = 0%), and intranasal (RR: 0.31, 95% CI: 0.24–0.40, *P* < 0.00001, I^2^ = 34%) routes significantly reduced the incidence of EA (Supplementary Table S5). There was no significant difference among the three subgroups (*P* = 0.23), indicating the beneficial effect of Dex across these common administration routes. However, the effect of the oral route (RR: 0.71, 95% CI: 0.30–1.69, *P* = 0.44) remains unclear, as only one study was included. The TSA outcome showed that cumulative Z-curves crossed the TSMB and reached the required IS for all three subgroups (Supplementary Fig. S3b, Table S4), further strengthening the robustness of the evidence.

### Foetal Exposure To Sevo Impaired Neuronal Migration in Mice

**Fig. 4:**
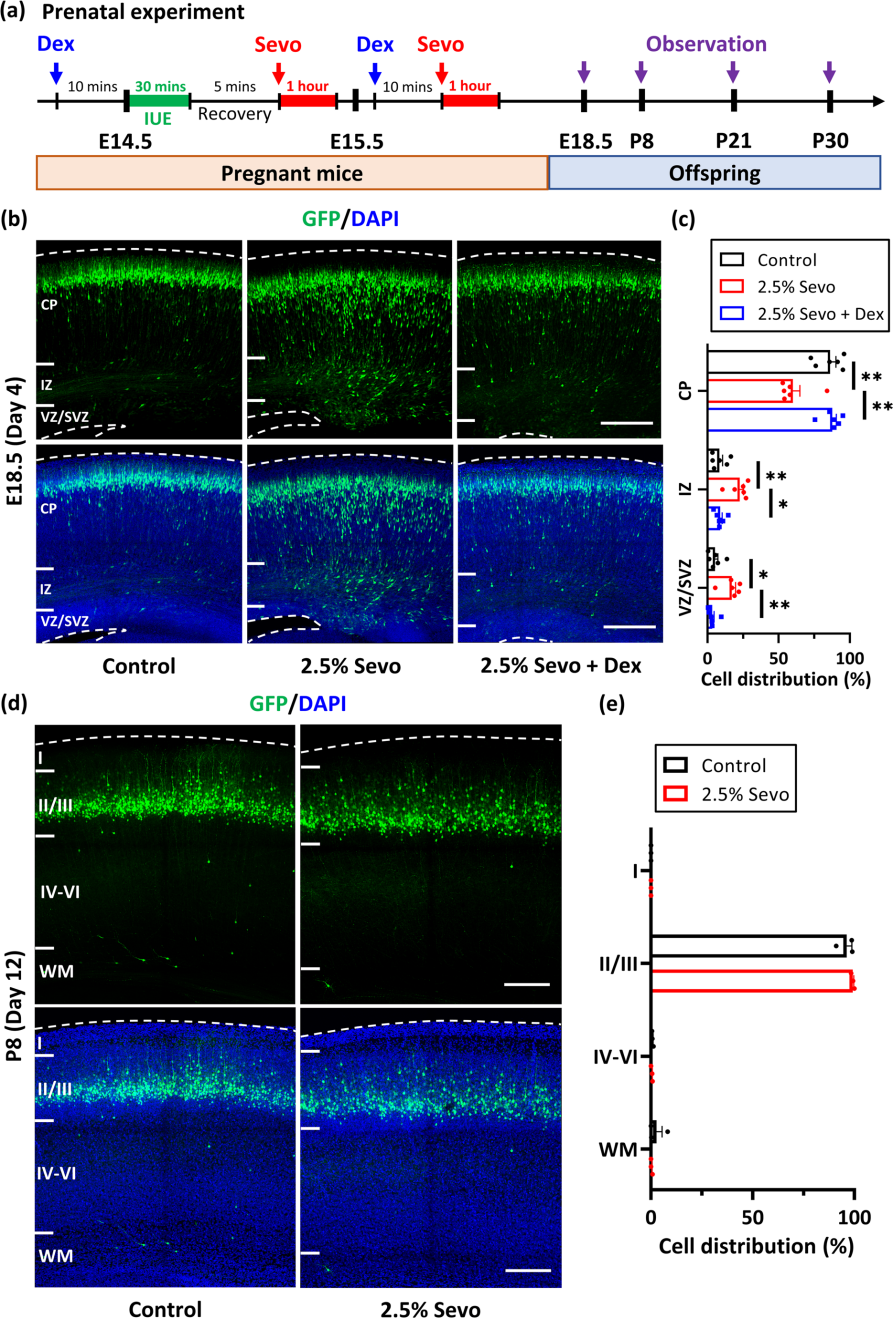
Dex prevents neuronal migration deficits caused by repeated Sevo exposure during pregnancy. (**a**) Schematic diagram of the prenatal experiment. IUE (green line) was performed on E14.5 pregnant mice, followed by general anaesthesia with Sevo (red arrows and lines) or pure oxygen for 1 h after recovering and on the next day (E15.5). Dex (blue arrows) or normal saline was injected intraperitoneally (IP) into the control, 2.5% Sevo, and 2.5% Sevo + Dex groups. Brains of the offspring were harvested at E18.5, P8, P21, or P30 for further observation (purple arrows). (**b**) Distributions of electroporated cells in the cerebral cortex at E18.5. Most GFP + cells (green) in the control group had migrated to the cortical plate (CP), while many GFP + cells in 2.5% Sevo group were delayed in the IZ and VZ/SVZ. Dex significantly restored migration in the 2.5% Sevo + Dex group. Scale bar = 200 μm. (**c**) Percentage of GFP + cells in the CP, IZ, and VZ/SVZ for each group (*n* = 6 mice per group). Error bars represent SEM. Kruskal-Wallis test, post-hoc: Two-stage linear step-up procedure of Benjamini, Krieger and Yekutieli. *: *p* < 0.05, **: *p* < 0.01. (**d**) Distribution of electroporated cells in the cerebral cortex at P8. GFP + cells in both control and 2.5% Sevo groups reached cortical layer II/III. Scale bar = 200 μm. (**e**) Percentage of GFP + cells in cortical layer I, II/III, IV-VI, and white matter (WM) for each group (*n* = 3 mice per group). Error bars represent SEM. Mann-Whitney test

To investigate the long-term effects of repeated gestational exposure to Sevo on cortical development, we established a prenatal experiment using a mouse model designed to mimic the anaesthetic condition during foetal surgery (Fig. 4a). Prior to anaesthesia, IUE was used to introduce green fluorescent protein (GFP)-expressing constructs into neural progenitor cells, thereby fluorescently labelling their progeny cells, primarily destined for the somatosensory cortex (Fig. 4b) (Huang et al. 2023). Subsequently, pregnant mice were exposed to 2.5% Sevo or 100% O_2_ (control) for 1 h on both E14.5 and E15.5, in accordance with previously established protocols (Chai et al. 2019, 2020). In control brains, most GFP + neurones successfully migrated to the cortical plate (CP) by E18.5 (Fig. 4b, c). In contrast, repeated exposure to 2.5% Sevo impaired neuronal migration, with many GFP + distributed in the intermediate zone (IZ) and the ventricular zone (VZ) (Fig. 4b, c). Interestingly, by postnatal day 8 (P8), the GFP + cells had migrated to cortical layer II/III, similar to the control group (Fig. 4d-e), suggesting a migration delay rather than a disruption.

**Fig. 5:**
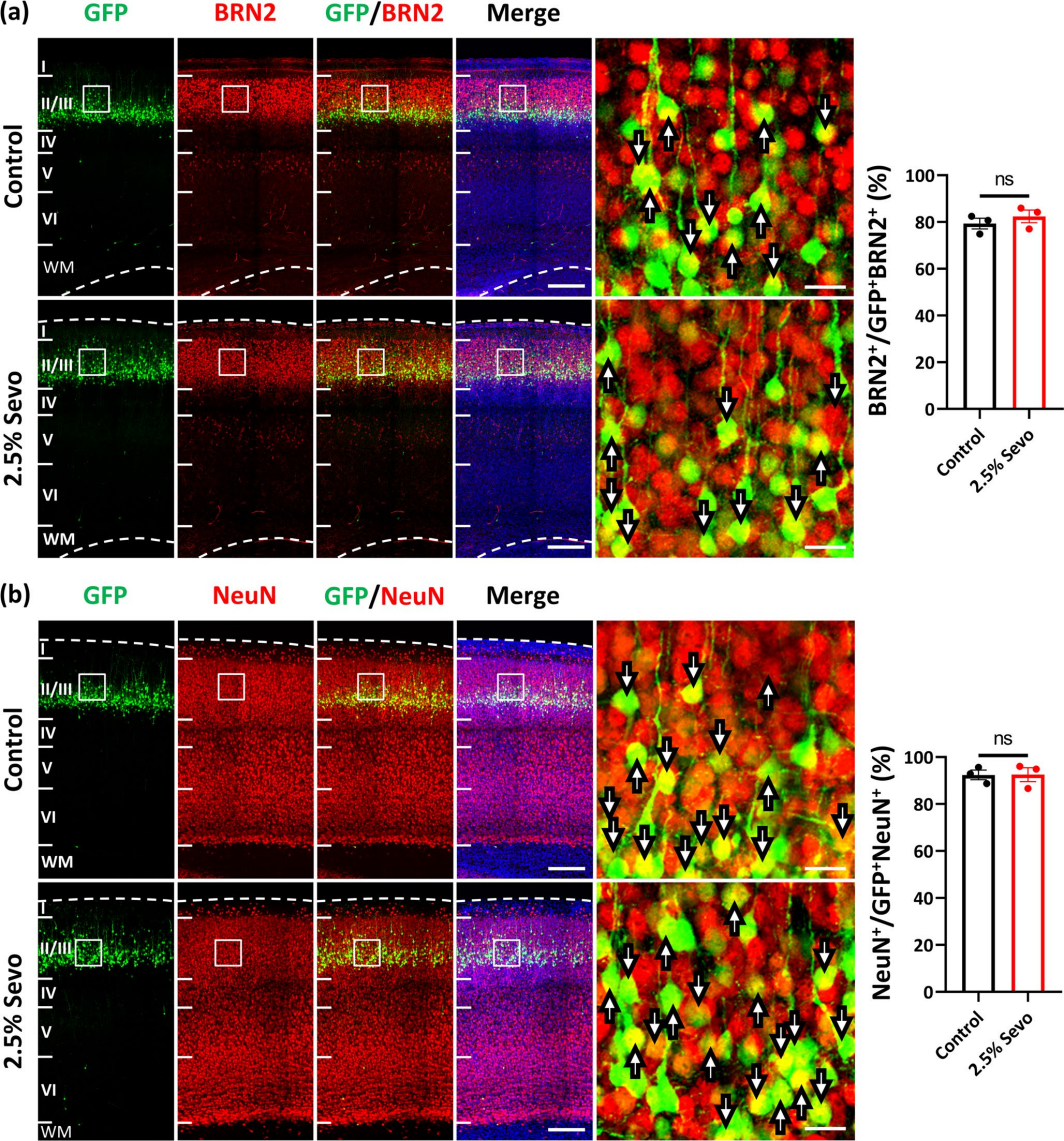
Expression of neuronal markers in neurones following in utero electroporation and repeated Sevo exposure. (**a**) Immunohistochemistry staining of electroporated cells (green) with BRN2 (red, cortical layer II/III marker) at P8. Most GFP + cells in the cortical layer II/III express BRN2 in both control (*n* = 3 mice) and 2.5% Sevo (*n* = 3 mice) groups. Right panel: High-magnification images (white boxes) show BRN2 expression in GFP + cells (arrows). Scale bar = 200 μm. Bar graph shows ratios of GFP + BRN2+/GFP + cells. (**b**) Similar staining for NeuN (red, post-mitotic neuronal marker). Most GFP + cells differentiate into neurones in both groups. Right panel: High-magnification images (white boxes) show NeuN expression in GFP + cells (arrows). Scale bar = 200 μm. Bar graph shows ratios of GFP + NeuN+/GFP + cells. Error bars represent SEM. Mann-Whitney test. ns: not significant

Furthermore, we monitored the differentiation of these GFP + cells in both groups and found that most of them expressed markers for postmitotic neurones (NeuN) and neurones in cortical layer II/III (BRN2) (Fig. 5). To evaluate whether prenatal Sevo exposure leads to prolonged pathological abnormalities, we analysed the dendritic structure of these neurones post-migration at P21 and P30 (Supplementary Fig. S4). Our results showed no significant differences in dendritic spine density and types between the control and 2.5% Sevo groups, except for the proportion of filopodia at P30 (Supplementary Fig. S4f). These results suggest that repeated exposure to 2.5% Sevo during pregnancy may not cause a prolonged disruption of dendritic spine development during adolescence.

### Dex Pretreatment Mitigated Sevo-Induced Neuronal Migration Delay

Given the reported neuroprotective properties of Dex during Sevo anaesthesia, we applied Dex pretreatment to pregnant mice before each Sevo anaesthesia session at E14.5 and 15.5. Remarkably, a single dose of Dex pretreatment alleviated the migration delay of developing neurones at E18.5 (Fig. 4a-c). Additionally, in the postnatal stage, Dex restored the proportion of filopodia spines in the 2.5% Sevo group to levels similar to the control group. These results suggest that the addition of Dex may provide a safer anaesthetic condition for pregnant individuals.

### Neonatal Sevo Anaesthesia Altered Dendritic Spine Density and Morphology

**Fig. 6:**
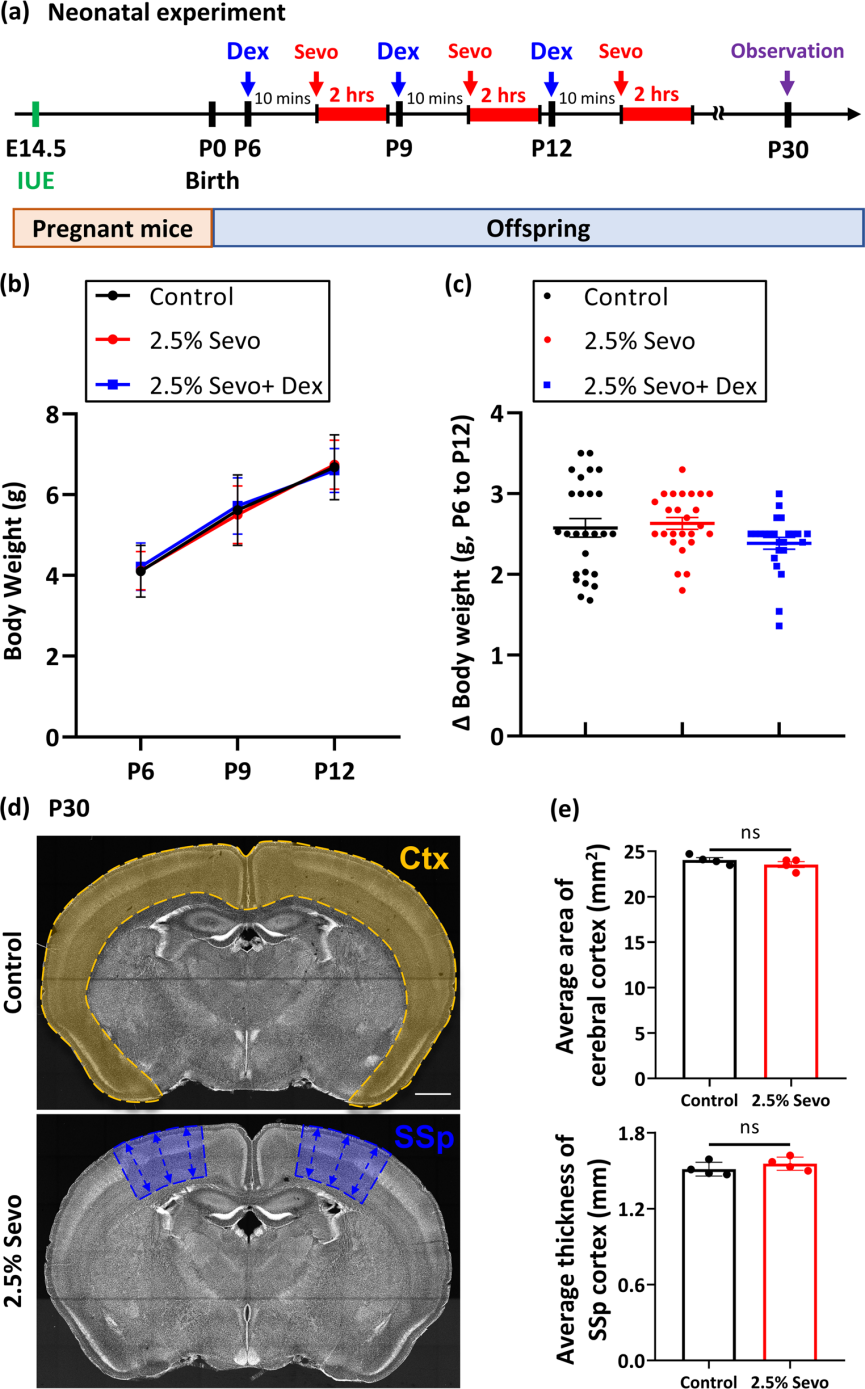
Impact of neonatal Sevo exposure on body weight and cortical structure in adolescent mice. (**a**) Schematic of the neonatal experiment. IUE (green line) was performed on E14.5 pregnant mice to label neurones with GFP. After birth, neonates were injected with Dex (blue arrows) or saline IP and anaesthetised with Sevo (red arrows and lines) or 60% oxygen for 2 h at P6, 9, and 12. Brains were analysed at P30. (**b**) Average body weight of neonatal mice from P6 to P12 in control (*n* = 26 mice), 2.5% Sevo (*n* = 25 mice), and 2.5% Sevo + Dex (*n* = 24 mice) groups. (**c**) Body weight changes between P6 and P12 for each group. (**d**) Representative coronal sections showing regions analysed for cerebral cortex (Ctx) and primary somatosensory cortex (SSp) morphology at P30. Scale bar = 1 mm (**e**) Area of Ctx and thickness of SSp in control (*n* = 4 mice) and 2.5% Sevo (*n* = 4 mice) groups. Error bars represent SEM. Mann-Whitney test. ns: not significant

General anaesthesia is a concern not only for foetuses during pregnancy but also for young children. To investigate the effects of repeated neonatal Sevo exposure, we administered 2.5% Sevo, 60% O_2_ (control), or 2.5% Sevo with Dex pretreatment (2.5% Sevo + Dex) to neonatal mice for 2 h on P6, 9, and 12 (Fig. 6a). We monitored the average body weight and body weight changes of mice in the control, 2.5% Sevo, and 2.5% Sevo + Dex groups and found no significant differences between them (Fig. 6b-c). These results indicate that the concentrations of Sevo and Dex used in the study did not affect the general development of these mice. Given the migratory deficits in cortical neurones found in the prenatal study, we examined the gross structure of the cerebral cortex by evaluating the cortical area and thickness at P30 in the neonatal study. We found no significant changes in mice exposed to Sevo compared to the control group (Fig. 6d-e).

**Fig. 7:**
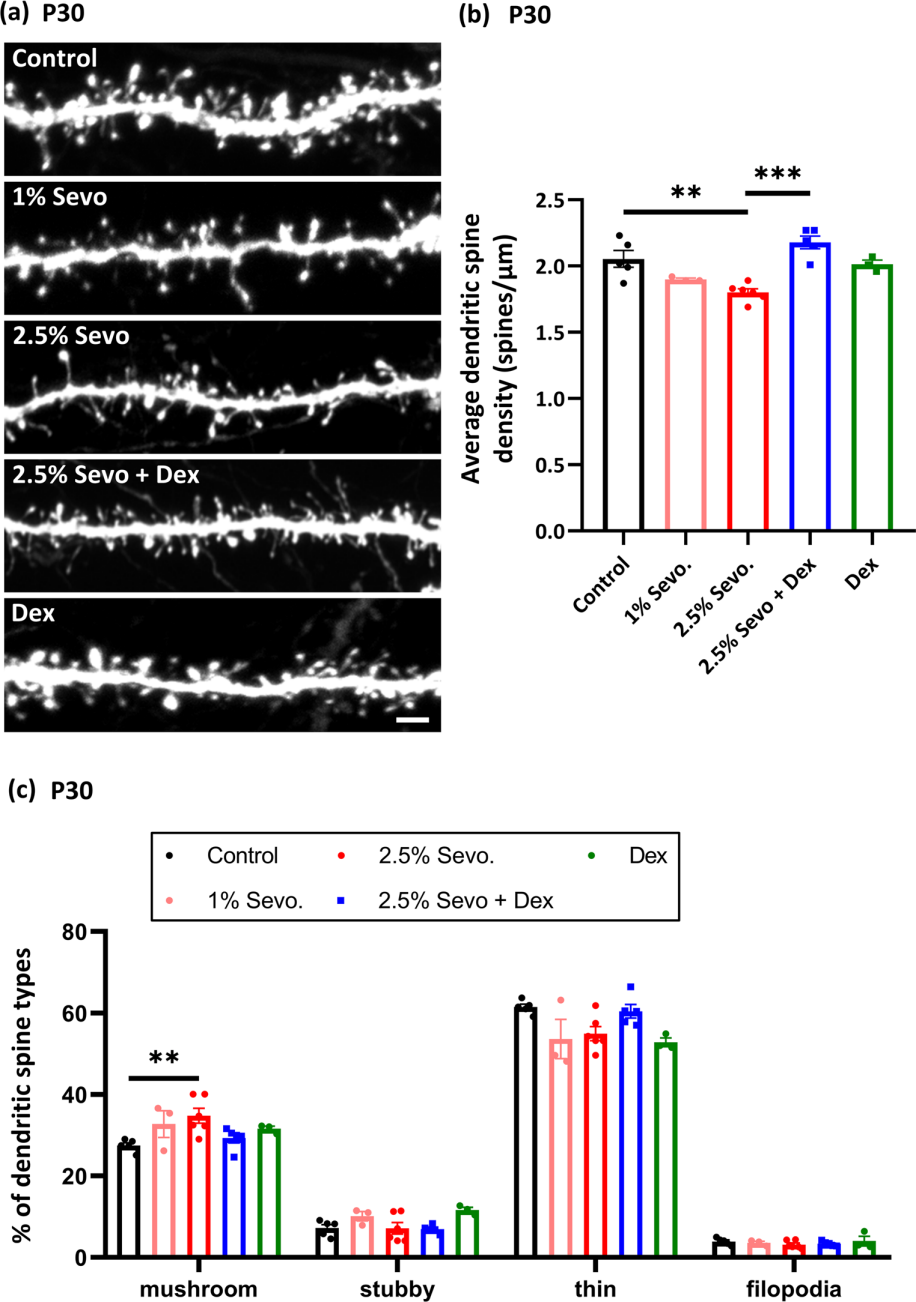
Dex pretreatment restores dendritic spine density in adolescents following neonatal Sevo anaesthesia. (**a**) Dendritic structures of GFP + neurones in the somatosensory cortex at P30. Significant reduction in dendritic spine density was observed in the 2.5% Sevo group compared to control. Dex pretreatment significantly increased dendritic spine density in the 2.5% Sevo + Dex group, similar to control. (**b**) Dendritic spine density statistics for control (*n* = 5 mice; 64 dendrites, 9225 spines), 1% Sevo (*n* = 3 mice; 34 dendrites, 4288 spines), 2.5% Sevo (*n* = 6 mice; 90 dendrites, 10218 spines), 2.5% Sevo + Dex (*n* = 5 mice; 55 dendrites, 8041 spines), Dex (*n* = 3 mice; 35 dendrites, 5115 spines) groups. (**c**) Proportion of each dendritic spine type (mushroom, stubby, thin, and filopodia) in analysed dendrites. The proportion of mushroom-type dendritic spines increased in the 2.5% Sevo group compared to control. Error bars represent SEM. Kruskal-Wallis test, post-hoc: Two-stage linear step-up procedure of Benjamini, Krieger and Yekutieli. **: *p* < 0.01, ***: *p* < 0.001

Recent studies have reported that repeated exposure to Sevo in neonates leads to global apoptosis, particularly in the neocortex (Areias et al. 2023), and negatively influences cognitive functions in rodent models (Apai et al. 2021). However, the impact of Sevo on synaptic architecture during cortical development remains unclear. Therefore, we investigated the effects of neonatal Sevo exposure (administered 3 times at P6, 9, and 12) on the morphologies of dendritic spines, crucial for the formation and complexity of the neuronal network. We monitored the morphologies of GFP + pyramidal neurones in layer II/III, previously electroporated with GFP at E14.5. We found that repeated neonatal exposure to both 1% and 2.5% Sevo significantly decreased dendritic spine density in adolescent mice at P30 (Figs. 6a and 7a-b). Additionally, we further analysed the morphological features of these dendritic spines and found a significant increase in mushroom-type spines in the 2.5% Sevo group compared to the control (Fig. 7c). The percentage of thin-type spines was also slightly decreased in both the 1% and 2.5% Sevo groups compared to the control, suggesting an alteration of spine types under Sevo anaesthesia.

### Dex Pretreatment Attenuated Changes in Dendritic Spines and Somatosensory Functions

To determine whether Dex pretreatment could mitigate changes in dendritic spine density and types, we evaluated dendritic spines in neonatal mice pretreated with Dex and subsequently exposed to 2.5% Sevo at P6, 9, and 12. Remarkably, Dex pretreatment effectively reversed the deficits caused by Sevo exposure, restoring dendritic spine density at P30 (Fig. 7a-b). Moreover, Dex pretreatment showed a trend towards restoring mushroom-type spines, although this was not statistically significant (Fig. 7c). Additionally, no significant differences were found in dendritic spine density and types between the control group and the groups receiving single dose of Dex, suggesting the potential for Dex in spine restoration (Fig. 7).

**Fig. 8:**
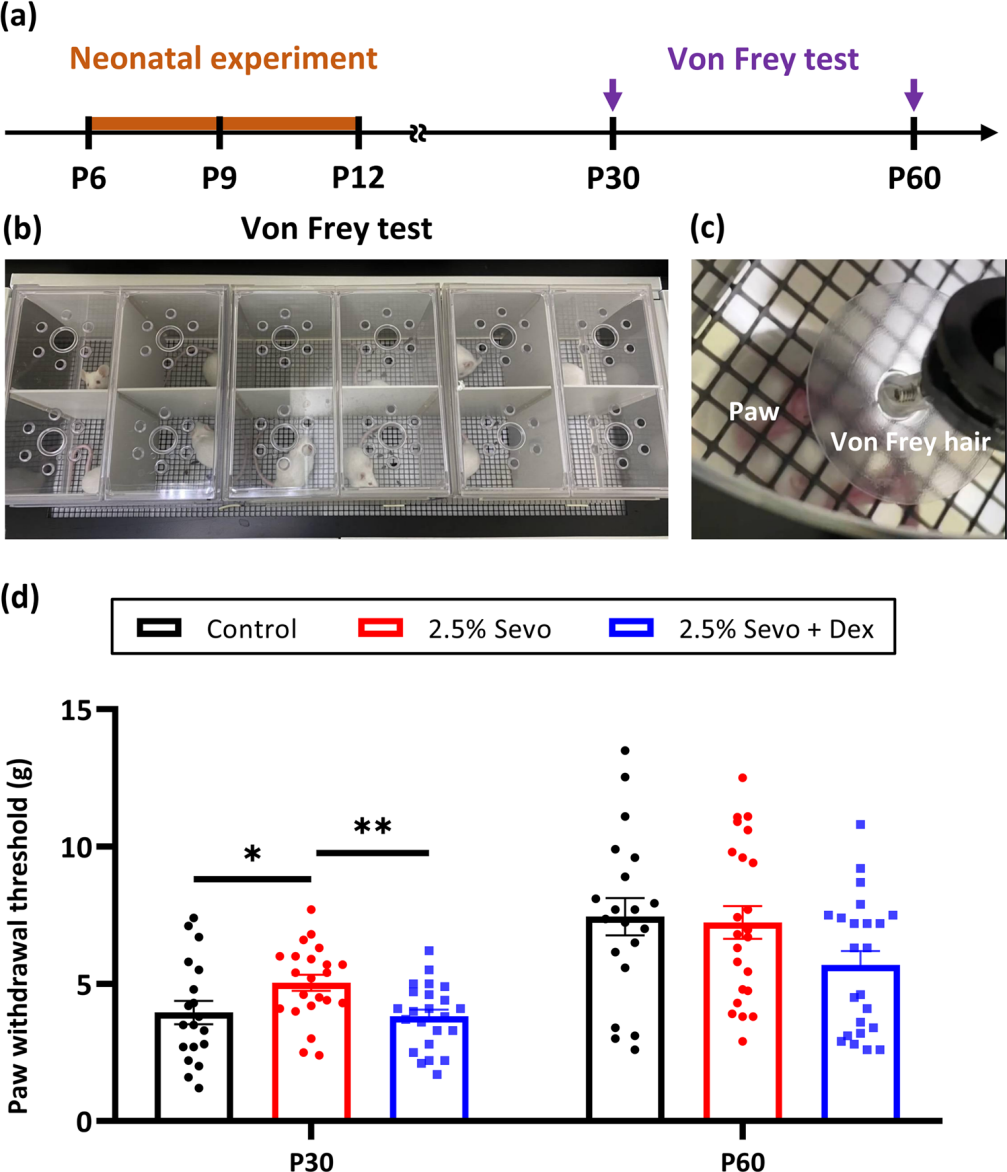
Dex pretreatment improves somatosensory function in adolescent mice. (**a**) Schematic of the neonatal experiment (brown line) and Von Frey (VF; purple arrows) test at P30 and P60. (**b**) VF test chamber setup. (**c**) Representative image mouse paw and Von Frey hair during the test. (**d**) Paw withdrawal threshold at P30 and P60 for control (P30: *n* = 19 mice, P60: *n* = 20 mice), 2.5% Sevo (P30: *n* = 22 mice, P60: *n* = 23 mice), and 2.5% Sevo + Dex (P30: *n* = 23 mice, P60: *n* = 23 mice) groups. Higher threshold in the 2.5% Sevo group at P30 indicates compromised somatosensory function. Dex pretreatment restores sensory and motor behaviour at P30. There is no significant difference at P60 among groups. Brown-Forsythe and Welch ANOVA tests, post-hoc: Two-stage linear step-up procedure of Benjamini, Krieger and Yekutieli. *: *p* < 0.05, **: *p* < 0.01

In addition to evaluating pathological changes in the developing cortex, we assessed the sensory and motor functions of the mice by the Von Frey (VF) test at 1 and 2 months of age (Fig. 8a-c). Similar to the pathological effects, animals exposed to 2.5% Sevo showed a higher threshold for paw withdrawal at P30, indicating compromised somatosensory function compared to the control group (Fig. 8d). Notably, Dex pretreatment markedly improved this function, aligning with its beneficial effects on dendritic spine development (Fig. 8d). However, no significant differences were observed among the control, 2.5% Sevo, and 2.5% Sevo + Dex groups in adult mice at P60 (Fig. 8d).

## Discussion

This study integrated a systematic review of clinical studies and a series of preclinical investigation to evaluate the effects of Dex premedication in the context of Sevo general anaesthesia on the developing brains. While our meta-analysis summarized the short-term benefits of Dex in reducing the incidence of EA, the preclinical component of this work revealed Dex’s potential long-term neuroprotective effects. In the clinical part of this study, we systematically reviewed clinical trials examining the combined use of Dex and Sevo in paediatric anaesthesia, with a specific focus on the incidence of EA in children under 8 years of age. To our knowledge, this may represent the first systematic review specifically targeting this high-risk population (Lee and Sung 2020; Urits et al. 2020). While various factors contribute to the postoperative EA, such as preoperative anxiety (Kain et al. 2004), the type of the procedure, and postoperative pain, the fast-acting nature of inhalational agents like Sevo and the age of the patient are critical risk factors.

Despite its drawbacks, Sevo remains indispensable in clinical practice, particularly for mask induction, even though more anaesthetic strategies, including total intravenous anaesthesia and regional blocks (Suresh et al. 2018), are integrated into pediatric anaesthesia. Dex is known to act on both adrenergic and nonadrenergic neurons in the locus coeruleus, leading to a sedative state similar to natural sleep (Weerink et al. 2017; Chamadia et al. 2020), in addition to its analgesic and sympatholytic effects. In recent years, Dex has gained prominence in paediatric anaesthetic practice for its potential neuroprotective effects and its ability to preserve neurocognitive function (Pan et al. 2016; Mahmoud et al. 2020). However, the limited numbers of longitudinal studies examining the association between early Dex exposure and long-term neurodevelopmental outcomes underscores a critical gap in our understanding. Our meta-analysis revealed that all common routes of Dex administration–intravenous, intranasal, and perineural–effectively reduced the incidence of EA following Sevo anaesthesia. Therefore, it is crucial to understand these negative effects and explore safer, more refined procedures for administering general anaesthesia in paediatric surgeries. More research into micro-connectivity or ultrastructure of the brain is warranted.

While our meta-analysis focused on the short-term effects of Dex in reducing EA, recent preclinical studies have also indicated the potential long-term neurotoxic effects of Sevo use during pregnancy and paediatric anaesthesia (Liang et al. 2023; Wang et al. 2024a; Cao et al. 2025; Chaki et al. 2025; Che et al. 2025; Yu et al. 2025). In our preclinical investigation, we used a mouse model of paediatric anaesthesia to demonstrate the distinct neurotoxic effects of repeated Sevo exposure at different developmental stages. Prenatal exposure to Sevo resulted in significant delays in neuronal migration, while postnatal exposure during the period of spinogenesis in childhood significantly impaired dendritic spine development in adolescence. These in vivo findings provide insights into the potential neuropathological consequences of Sevo exposure during critical periods of brain development. To emphasise the importance of refining general anaesthesia procedures in paediatrics, we also explored the neuroprotective properties of Dex against the adverse effects induced by Sevo, both during the prenatal and neonatal stages. Our results demonstrate that Dex exhibits superior beneficial effects, particularly in mitigating migratory deficits and preventing dendritic spine loss. Although there is currently a lack of clinical studies examining the long-term neuroprotective effects of Dex in paediatric populations, our findings suggest that Dex holds promise as a strategy to enhance the safety of anaesthesia protocols for vulnerable patients in early life.

During surgical operation, maintaining an adequate minimum alveolar concentration (MAC) of inhaled anaesthetic is crucial to ensure sufficient depth of anaesthesia to mitigate sensory and nociceptive stimuli. The specific MAC concentration for mouse models was determined based on the type of anaesthetic agents used, the mouse strain, and the age of the mice (Cesarovic et al. 2010). For ICR mice, the MAC of Sevo has been reported to be 3.22% in adults (Ichinose et al. 1998). In our prenatal and neonatal studies, we used relatively low concentrations of 1% (~ 0.31 MAC) or 2.5% (~ 0.78 MAC). Prenatal exposure to Sevo at a concentration of 0.78 MAC, with a cumulative duration of two hours, impaired neuronal migration in embryonic brains. Similarly, neonatal exposure to Sevo at 0.31 or 0.78 MAC, administered three times with a cumulative duration of 6 h, led to significant neuronal abnormalities during adolescence. These findings raised concerns about the extent of Sevo exposure in developing brains and emphasise the urgent need for modified protocols that use reduced Sevo concentration or incorporate neuroprotective agents in clinical practice.

Neuronal migration plays a crucial role in the development of the cerebral cortex and is closely associated with cognitive functions. Disruptions in these migratory processes often lead to a spectrum of neuronal migration disorders, including lissencephaly and subcortical heterotopia (Copp and Harding 1999). Previous studies have reported that maternal exposure to Sevo affects neuronal migration in mice, suggesting that multiple exposures (at least twice), each lasting 2 h per day (~ 0.77 MAC), cause a delay in neuronal migration in the foetal brains at E18.5 (Chai et al. 2019, 2020). In our study, we showed that migrating cortical neurones were temporarily delayed in the IZ and VZ/SVZ at E18.5 in ICR mice exposed to Sevo at a comparable MAC (~ 0.78 MAC), but with only half the exposure time (1 h per day) compared to the previous study (Chai et al. 2019, 2020). Additionally, our investigation extended beyond the prenatal stage, following the migrating neurones up to P8. We observed that these initially delayed neurones ultimately reached their destinations in the outer cortical layers and maintained their expected neuronal identity. These findings align with clinical observations that children who underwent foetal operation did not exhibit significant changes in the macrostructure of the cortex. Together, our data suggest that repeated maternal exposure to Sevo may result in a transient delay in neuronal migration in foetal brains without causing long-term cortical malformations in the postnatal period.

In the mammalian brain, cortical neurones begin developing dendrites and axons after migration, establishing connections with neighbouring neurones to form the complex neural network essential for information processing (Berry and Nedivi 2017). The equilibrium of spine dynamics is crucial for regulating the plasticity necessary for learning and memory (Berry and Nedivi 2017). In this study, we specifically investigated the dendritic spine density and morphology of layer II/III pyramidal neurones in adolescent mice that experienced multiple Sevo exposures in gestation or childhood. We found that repeated Sevo exposures in childhood resulted in decreased dendritic spine density and an abnormal proportion of spine types in the somatosensory cortex in adolescence. Our findings are consistent with previous studies showing that repeated Sevo exposure during neonatal stages decreased the dendritic spine density of hippocampal neurones in both adult mice (Ji et al. 2015; Zeng et al. 2022) and rats (Amrock et al. 2015; Ju et al. 2016; Xu et al. 2023). The decrease in dendritic spine density could be a consequence of reduced spine formation or increased spine elimination.

Although both EA and altered behavioral responses in the Von Frey test may reflect disruptions in neural function following anaesthetic exposure, their underlying mechanisms are likely distinct. EA is typically a transient, acute behavioral manifestation observed during the early recovery phase from anaesthesia and is thought to involve perturbations in cortical arousal and noradrenergic signaling. In contrast, performance in the Von Frey test, which assesses mechanical nociception thresholds, may reflect long-term alterations in sensory or motor processing arising from structural changes in the somatosensory cortex. While abnormal sensitization could potentially contribute to both EA and altered Von Frey responses, our findings suggest that the latter is more closely associated with dendritic spine deficits resulting from repeated neonatal exposure to Sevo. Notably, Dex pretreatment restored both dendritic spine density and somatosensory function, implying a neuroprotective effect at the synaptic level. However, given the complex nature of these behavioral outcomes, further investigation is warranted to delineate the specific neural circuits and cellular mechanisms that link anaesthetic exposure to both immediate and long-term functional impairments.

While both the reduction of EA and neuroprotection effects of Dex may involve modulation of noradrenergic signalling, they likely engage distinct mechanisms. Dex’s ability to attenuate EA appears to arise primarily from its sedative properties and attenuation of stress responses, whereas its neuroprotective actions are thought to be mediated through anti-inflammatory and anti-apoptotic pathways. Preclinical studies have consistently shown that exposure to Sevo induces neuroinflammation and microglial overactivation in the developing mouse brain (Xu et al. 2023). Excessive engulfment of dendritic spines by activated microglia may contribute to the loss of these structures (Stein and Zito 2019; Farzinpour et al. 2022; Xu et al. 2023). Furthermore, Dex has been considered as a potential modulator of inflammation, with many studies demonstrating its ability to reduce neuroinflammation by inhibiting the activation of microglia and the pro-inflammatory response of astrocytes (Chen et al. 2022). Therefore, we propose that Dex may restore dendritic spine density by suppressing microglia-mediated dendritic spine elimination resulting from Sevo-induced microglia activation. However, further investigations are needed to examine the molecular mechanism underlying Dex’s influence on dendritic spine dynamics. Additionally, comparative studies assessing Dex’s neuroprotective efficacy against other anaesthetic adjuvants, such as propofol or ketamine, may yield valuable insights.

In conclusion, the current study represents the first comprehensive exploration of the impacts of repeated Sevo exposure during gestational and neonatal stages on neuronal development in the developing cortex. Our meta-analysis demonstrated the short-term benefits of Dex in reducing the incidence of EA. Using a mouse model, we identified neuropathological changes correlated with cognitive impairments observed in paediatric postoperative cases. Moreover, our findings underscore the potential benefits of Dex in mitigating Sevo-induced neurotoxicity. Future research should focus on more extensive investigations into the therapeutic efficacy and underlying mechanisms of Dex to provide a deeper understanding of these interventions. Further clinical trials are essential to validate its long-term neuroprotective benefits and determine optimal dosing strategies prior to widespread clinical implementation.

## Limitation of this Study

This study integrates clinical and preclinical approaches to investigate the neurodevelopmental effects of sevoflurane (Sevo) exposure and the potential neuroprotective role of dexmedetomidine (Dex). However, several limitations must be acknowledged.

First, while our meta-analysis focuses on the short-term clinical outcome of EA, the preclinical component investigates long-term neurodevelopmental alterations–namely, delays in neuronal migration and dendritic spine deficits. EA is a transient behavioral phenomenon, and its pathophysiology may not be directly recapitulated by these structural changes. Therefore, a translational gap exists between our clinical and preclinical findings. Moreover, our preclinical model, which uses IUE, is not designed to simulate a specific surgical scenario but rather to serve as a robust platform for labeling and tracking neuronal populations to study developmental processes in response to anaesthetic exposure.

Second, the Von Frey test was performed 18 days after Sevo and Dex exposure. Given the pharmacokinetics of Dex, it is unlikely that short-term sedation effects would persist long enough to influence nociceptive thresholds at this time point. Although a Dex-only control group was not included in the behavioral assessment, our dendritic spine analysis demonstrates that Dex alone did not alter spine density in the somatosensory cortex, a key structural correlate of synaptic integrity. Nonetheless, the Von Frey test involves both sensory and motor components, and accurate measurement of mechanical pain thresholds depends on preserved motor function. Therefore, potential locomotor deficits could theoretically confound the interpretation. While our study did not directly assess motor performance, recent reports using similar exposure paradigms found that repeated Sevo administration did not affect general locomotor activity as evaluated by the open field test (Wang et al. 2024b; Cao et al. 2025). However, mild impairments in fine motor coordination were observed in the rotarod test following repeated neonatal Sevo exposure, likely resulting from disrupted myelination in the corpus callosum (Cao et al. 2025; Che et al. 2025).

Third, 100% oxygen was used during prenatal anesthesia, primarily due to increased maternal mortality observed when using 60% oxygen following IUE. While this approach ensured maternal and foetal survival, hyperoxia itself may contribute to neurotoxicity via oxidative stress, microglial activation, and white matter damage. Although our study does not directly assess Dex’s ability to mitigate hyperoxia-induced injury, its known anti-inflammatory and anti-apoptotic effects suggest that it may confer protection in this context. Future studies should specifically address this possibility to disentangle the effects of Sevo and oxygen.

Fourth, while our analysis focused on the somatosensory cortex, future investigations should extend to additional brain regions, such as the medial prefrontal cortex (mPFC), which is implicated in executive function and social cognition and can be targeted using our IUE protocol. Previous studies have shown that Sevo alters spine density and synaptic transmission in mPFC layer V pyramidal neurons (Briner et al. 2010; Chung et al. 2017b). Moreover, behavioral paradigms assessing cognitive function, such as the Morris Water Maze, could be employed to determine whether Dex-mediated structural preservation translates into functional rescue.

Finally, while our data highlight both short- and long-term benefits of Dex, its safety profile in neonates remains an area of concern. Dex has been associated with hemodynamic side effects—including bradycardia, hypotension, and transient hypertension—particularly in the neonatal population (Portelli et al. 2024). Long-term neurodevelopmental outcomes following Dex exposure are not well characterized. Ongoing trials, such as the DICE trial (Dexmedetomidine Use in Infants Undergoing Cooling Due to Neonatal Encephalopathy), are expected to provide crucial data regarding the long-term safety and efficacy of Dex in neonates (Baserga et al. 2021).

## Supplementary Information

Below is the link to the electronic supplementary material.

Supplementary Material 1 (PDF 3.01 MB)

## Data Availability

Data is provided within the manuscript or supplementary information files.
