# Supplementary material for: Dexmedetomidine Mitigates Sevoflurane-Induced Neurodevelopmental Effects in Paediatric Anaesthesia: A Meta-Analysis and Preclinical Study
Source: J Neuroimmune Pharmacol. 2026 Jan 7;21(1):5. doi: 10.1007/s11481-025-10273-8 (PMC12779744; doi:10.1007/s11481-025-10273-8)
Supplement: Supplementary file 1 — Supplementary Material 1 (PDF 3.01 MB) [file 11481_2025_10273_MOESM1_ESM.pdf]

Table S1

Table S1. Detailed search strategy for each database, including MEDLINE, Cochrane, and Embase.

Search number	Query	Results found
**MEDLINE**		
Recent queries in Pubmed on Sep 12, 2023		
#1	"infant"[MeSH Term#s]	1,255,295
#2	"Child, Preschool"[MeSH Terms]	1,000,757
#3	((((“Child”[Mesh]) OR (“Infant”[Mesh])) OR (child*[Title/Abstract])) OR (infant*[Title/Abstract])) OR (pediatric*[Title/Abstract])	3,404,130
#4	#1 OR #2 OR #3	3,404,130
#5	sevoflurane[MeSH Terms]	7,348
#6	(((((sevoflurane) OR (Fluoromethyl-2,2,2-trifluoro-1-(trifluoromethyl)ethyl Ether)) OR (Fluoromethyl Hexafluoroisopropyl Ether)) OR (Sevorane)) OR (Ultane)) OR (BAX 3084)	11,161
#7	#5 OR #6	11,161
#8	precedex[MeSH Terms]	5,743
#9	((((dexmedetomidine) OR (precedex)) OR (MPV1440)) OR (Dexmedetomidine Hydrochloride)) OR (Hydrochloride, Dexmedetomidine)	8,977
#10	#8 OR #9	8,977
#11	Emergence Delirium[MeSH Terms]	844
#12	((((((((((((((((((Delirium, Emergence) OR (Emergence Agitation)) OR (Agitation, Emergence)) OR (Agitations, Emergence)) OR (Post-Operative Delirium)) OR (Delirium, Post-Operative)) OR (Post Operative Delirium)) OR (Postanesthetic Excitement)) OR (Excitement, Postanesthetic)) OR (Anesthesia Emergence Delirium)) OR (Delirium, Anesthesia Emergence)) OR (Emergence Delirium, Anesthesia)) OR (Postoperative Delirium)) OR (Delirium, Postoperative)) OR (Agitated Emergence)) OR (Emergence, Agitated)) OR (Emergence Excitement)) OR (Excitement, Emergence)) OR (delirium)) OR (agitation)	48,728
#13	#11 OR #12	48,728
#14	#4 AND #7 AND #10 AND #13	99
**Cochrane**		
Recent queries in Cochrane on Sep 12, 2023		
#1	MeSH descriptor: [Child, preschool] explode all trees	39,489
#2	MeSH descriptor: [Infant] explode all trees	45,034
#3	MeSH descriptor: [Pediatrics] explode all trees	1,044
#4	(child* or infant* or pediatric*):ti,ab,kw (Word variations have been searched)	235,074
#5	#1 OR #2 OR #3 OR #4	235,076
#6	MeSH descriptor: [Sevoflurane] explode all trees	2,698
#7	(sevoflurane) OR (Fluoromethyl Hexafluoroisopropyl Ether) OR (BAX 3084) OR (Sevorane) OR (Ultane)	7,240
#8	#6 OR #7	7,240
#9	MeSH descriptor: [Dexmedetomidine] explode all trees	2,942
#10	(dexmedetomidine) OR (precedex) OR (MPV1440) OR (Dexmedetomidine Hydrochloride) OR (Hydrochloride, Dexmedetomidine)	8,510
#11	#9 OR #10	8,510
#12	MeSH descriptor: [Emergence Delirium] explode all trees	385
#13	((Emergence OR Post-Operative OR Post operative OR Postoperative OR Postanesthetic OR Anesthesia Emergence) NEAR/4 (delirium OR agitation OR agitations OR agitated OR Excitement)):ti,ab,kw	3,369
#14	#12 OR #13	3,369
#15	#5 AND #8 AND #11 AND #14	133

Table S1

EMBASE

Recent queries in EMBASE on Sep 12, 2023

#1	'Child, Preschool'/exp	718,162
#2	'infant'/exp	1,325,097
#3	'pediatric'/exp	41
#4	preschool AND child*:ti,ab	414,295
#5	infant*:ti,ab	591,344
#6	pediatric*:ti,ab	614,577
#7	'child'/exp	3,486,909
#8	child*:ti,ab	2,264,522
#9	((((((((preschool child) OR (children, preschool)) OR (Preschool Children)) OR (infant)) OR (toddler)) OR (early childhood)) OR (little kid)) OR (newborn)) OR (neonate)) OR (pediatric)	2,884,743
#10	#1 OR #2 OR #3 OR #4 OR #5 OR #6 OR #7 OR #8 OR #9	4,555,464
#11	'sevoflurane'/exp	26,954
#12	sevoflurane OR (fluoromethyl AND hexafluoroisopropyl AND ether) OR (bax AND 3084) OR sevorane OR ultane	27,883
#13	#11 OR #12	27,883
#14	'precedex'/exp	18,658
#15	dexmedetomidine OR precedex OR mpv1440 OR (dexmedetomidine AND hydrochloride) OR (hydrochloride, AND dexmedetomidine)	19,237
#16	#14 OR #15	19,237
#17	'emergence agitation'/exp	1,008
#18	((emergence OR 'post-operative' OR 'post operative' OR postoperative OR postanesthetic OR 'anesthesia emergence') NEAR/3 (delirium OR agitation OR agitations OR agitated OR excitement)):ti,ab,kw	6,240
#19	delirium:ti,ab	33,885
#20	excitement:ti,ab	7,435
#21	agitation*:ti,ab	32,784
#22	agitated:ti,ab	8,789
#23	#17 OR #18 OR #19 OR #20 OR #21 OR #22	72,896
#24	#10 AND #13 AND #16 AND #23	220

Table S2

Table S2. List of excluded studies and reasons for exclusion.

Reason	Numbers	References
Not sevoflurane-based	3	[1-3]
Not dexmedetomidine-based	1	[4]
Non-english-based	9	[5-13]
Age not included	25	[14-38]
Full-article not retrieved	12	[39-50]
Both experimental group and control group received equal dosage of dexmedetomidine	2	[51,52]

Reference in Table S2

1. Devroe S, Devriese L, Debuck F, et al. Effect of xenon and dexmedetomidine as adjuncts for general anesthesia on postoperative emergence delirium after elective cardiac catheterization in children: study protocol for a randomized, controlled, pilot trial. Trials 2020;21:310.
2. Cheng X, Huang Y, Zhao Q, Gu E. Comparison of the effects of dexmedetomidine-ketamine and sevoflurane-sufentanil anesthesia in children with obstructive sleep apnea after uvulopalatopharyngoplasty: An observational study. J Anaesthesiol Clin Pharmacol 2014;30:31-5.
3. Hanafy MA, El-Zahaby HM, Swellam AM. The effect of dexmedetomidine on the emergence agitation associated with desflurane anaesthesia in children. Egyptian Journal of Anaesthesia 2004;20:135-40.
4. Long MHY, Lim EHL, Balanza GA, Allen JC, Purdon PL, Bong CL. Sevoflurane requirements during electroencephalogram (EEG)-guided vs standard anesthesia Care in Children: A randomized controlled trial. Journal of Clinical Anesthesia 2022;81.
5. Yan X, Xu Y, Lin N. Influence of intranasal dexmedetomidine premedication on general anesthesia effect and safety in pediatric patients undergoing monocular fundus examination. Adverse Drug Reactions Journal 2020;22:289-94.
6. Peng W, Tu W, Liao J, Lan Z. Optimal dosage of dexmedetomidine on prevention of agitation induced by sevoflurane anesthesia in children. Chinese Journal of Clinical Pharmacology and Therapeutics 2020;25:75-80.
7. Cong-Na Z, Juan F, Zhen X, Xian M, Fang W. Effects of sevoflurane combined with dexmedetomidine on restlessness during general anesthesia in children with ophthalmology. International Eye Science 2019;19:1945-9.
8. Chai DD, Ji J. [Effect of dexmedetomidine combined with sevoflurane for general anesthesia during dental treatment in pediatric patients]. Shanghai Kou Qiang Yi Xue 2018;27:85-8.
9. Cao X, Zhao L, Liu X, et al. Sedative effect of non-intravenous administration dexmedetomidine in pediatric patients underwent lower abdomen and limb surgery. Journal of Jilin University Medicine Edition 2018;44:388-93.
10. Lin L, Yueming Z, Meisheng L, Jiexue W, Yang J. [Effect of dexmedetomidine on emergence agitation after general anesthesia in children undergoing odontotherapy in day-surgery operating room]. Hua Xi Kou Qiang Yi Xue Za Zhi 2017;35:613-7.
11. Wang JX, Shi SZ, Li JH, Fei X, Li MM. Application effects of dexmedetomidine combined with sevoflurane for anesthesia in short operations in children with burn injury. Medical Journal of Chinese People's Liberation Army 2016;41:254-7.
12. Di M, Huang C, Chen F, et al. [Effect of single-dose dexmedetomidine on recovery profiles after sevoflurane anesthesia with spontaneous respiration in pediatric patients undergoing cleft lip and palate repair]. Zhonghua Yi Xue Za Zhi 2014;94:1466-9.
13. Kawaai H, Sato J, Watanabe M, et al. Dexmedetomidine for postoperative management after sevoflurane anesthesia in children. Journal of Japanese Dental Society of Anesthesiology 2008;36:269-77.
14. Shama AAA, Elsayed AA, Albraithen AA, Arafa SK. Effect of Dexmedetomidine, Dexamethasone, and Ondansetron on Postoperative Nausea and Vomiting in Children Undergoing Dental Rehabilitation: A Randomized Controlled Trial. Pain Physician 2023;26:1-11.
15. Omara AF, Elzohry AA, Abdelrahman AF. Comparison of Caudal Block and Dexmedetomidine Infusion in Pediatric Patients Undergoing Hypospadias Repair Surgery: A Prospective, Randomized, Double-blinded Clinical Study. Anesthesiology and Pain Medicine 2023;13.
16. Sadeghi A, Razavi SS, Eghbali A, Mahdavi SA, Kimia F, Panah A. The Comparison of the Efficacy of Early versus Late Administration of Dexmedetomidine on Postoperative Emergence Agitation in Children Undergoing Oral Surgeries: A Randomized Clinical Trial. Iranian Journal of Medical Sciences 2022;47:25-32.
17. Lei DX, Wu CJ, Wu ZY, Wang LY, Zhao Q, She YJ. Efficacy of different doses of intranasal dexmedetomidine in preventing emergence agitation in children with inhalational anaesthesia: A prospective randomised trial. European Journal of Anaesthesiology 2022;39:858-67.
18. Amer GF, Abdallah MY. Dexmedetomidine versus propofol for prevention of emergence delirium in pediatric cataract surgery: Double blinded randomized study. Egyptian Journal of Anaesthesia 2022;38:300-4.
19. Ramlan AAW, Mahri I, Firdaus R, Sugiarto A. Comparison of Efficacy of Premedication between Dexmedetomidine and Midazolam Intranasal for the Prevention of Emergence Delirium in Children Undergoing Ophthalmic Surgery. Turk J Anaesthesiol Reanim 2021;49:439-44.
20. Ramachandran A, Palanisamy N, Vidya MV, Zachariah M, George SK, Ranjan RV. Comparison of dexmedetomidine in two different doses on emergence agitation in children under sevoflurane anaesthesia: A double-blind randomised controlled trial. Indian J Anaesth 2021;65:519-24.
21. Oriby ME, Elrashidy A. Comparative Effects of Total Intravenous Anesthesia with Propofol and Remifentanil Versus Inhalational Sevoflurane with Dexmedetomidine on Emergence Delirium in Children Undergoing Strabismus Surgery. Anesth Pain Med 2021;11:e109048.
22. Chen G, Yang J, Chen Q, Liu D. Ultrasonic Image Restoration Algorithm for Prevention of Nervous Disorders during the Recovery Period of Patients Receiving Sevoflurane Anesthesia. J Healthc Eng 2021;2021:6124346.
23. Cho EA, Cha YB, Shim JG, Ahn JH, Lee SH, Ryu KH. Comparison of single minimum dose administration of dexmedetomidine and midazolam for prevention of emergence delirium in children: a randomized controlled trial. J Anesth 2020;34:59-65.
24. Gullu HA, Orhon ZN, Acar GO, Ozdamar OI, Celik MG. Assessment of the success of drugs to reduce the emergence agitation in children following adenotonsillectomy. Kuwait Medical Journal 2019;51:21-6.
25. Begum U, Singh PR, Naithani B, Singh V, Singh GP, Tiwari T. Dexmedetomidine as Bolus or Low-dose Infusion for the Prevention of Emergence Agitation with Sevoflurane Anesthesia in Pediatric Patients. Anesth Essays Res 2019;13:57-62.

Table S2

26. Park SJ, Shin S, Kim SH, et al. Comparison of dexmedetomidine and fentanyl as an adjuvant to ropivacaine for postoperative epidural analgesia in pediatric orthopedic surgery. Yonsei Medical Journal 2017;58:650-7.
27. Kavya Prabhu M, Mehandale SG. Comparison of oral dexmedetomidine versus oral midazolam as premedication to prevent emergence agitation after sevoflurane anaesthesia in paediatric patients. Indian Journal of Anaesthesia 2017;61:131-6.
28. Jia ZM, Hao HN, Huang ML, Ma DF, Jia XL, Ma B. Influence of dexmedetomidine to cognitive function during recovery period for children with general anesthesia. Eur Rev Med Pharmacol Sci 2017;21:1106-11.
29. Bedirli N, Akçabay M, Emik U. Tramadol vs dexmedetomidine for emergence agitation control in pediatric patients undergoing adenotonsillectomy with sevoflurane anesthesia: prospective randomized controlled clinical study. BMC Anesthesiol 2017;17:41.
30. Bai Y, Yu H, Wang M, et al. Effect of intraoperative application of dexmedetomidine on early postoperative cognitive function and serum brain-derived neurotrophic factor (BDNF) in children undergoing tonsillectomy. International Journal of Clinical and Experimental Medicine 2016;9:8482-9.
31. Xiao CL, Zhao T, Zhang YP. Optimal dosage of three dexmedetomidine doses on prevention of agitation induced by sevoflurane anesthesia in children. Journal of Dalian Medical University 2015;37:379-81 and 84.
32. Soliman R, Alshehri A. Effect of dexmedetomidine on emergence agitation in children undergoing adenotonsillectomy under sevoflurane anesthesia: A randomized controlled study. Egyptian Journal of Anaesthesia 2015;31:283-9.
33. Liu Y, Kang DL, Na HY, et al. Consequence of dexmedetomidine on emergence delirium following sevoflurane anesthesia in children with cerebral palsy. Int J Clin Exp Med 2015;8:16238-44.
34. Mizrak A, Karatas E, Saruhan R, et al. Does dexmedetomidine affect intraoperative blood loss and clotting tests in pediatric adenotonsillectomy patients? Journal of Surgical Research 2013;179:94-8.
35. Gupta N, Rath GP, Prabhakar H, Dash HH. Effect of intraoperative dexmedetomidine on postoperative recovery profile of children undergoing surgery for spinal dysraphism. J Neurosurg Anesthesiol 2013;25:271-8.
36. Meng QT, Xia ZY, Luo T, et al. Dexmedetomidine reduces emergence agitation after tonsillectomy in children by sevoflurane anesthesia: a case-control study. Int J Pediatr Otorhinolaryngol 2012;76:1036-41.
37. Akin A, Bayram A, Esmaoglu A, et al. Dexmedetomidine vs midazolam for premedication of pediatric patients undergoing anesthesia. Paediatr Anaesth 2012;22:871-6.
38. Özcengiz D, Gunes Y, Ozmete O. Oral melatonin, dexmedetomidine, and midazolam for prevention of postoperative agitation in children. J Anesth 2011;25:184-8.
39. Wu XL, Peng B, Shan CJ, Liu JJ, Zhang FC. The influence of dexmedetomidine on the emergence agitation of pediatric patients after the operations of sense organs under general anesthesia using sevoflurane. Minerva Pediatr (Torino) 2022;74:144-50.
40. Umar M, Ashfaq A, Zulfiqar H, Khan MA, Aziz HMI, Shabbir R. Assess the efficacy of 1 g/kg oral dexmedetomidine (dex) as a premedication in children experiencing dental treatments. NeuroQuantology 2022;20:4010-22.
41. Qiu J, Wang YY. The effects of different doses of alfentanil combined with dexmedetomidine during adenoidectomy in children with general anesthesia. Chinese Journal of New Drugs 2022;31:2257-61.
42. Endigeri A, Kalashetty M, Channappagoudar R, et al. COMPARISON OF NEBULIZED DEXMEDETOMIDINE VERSUS COMBINATION OF NEBULIZED KETAMINE AND MIDAZOLAM FOR PREMEDICATION IN PEDIATRIC PATIENTS UNDERGOING ELECTIVE SURGERY-A RANDOMIZED DOUBLE BLINDED STUDY. European Journal of Molecular and Clinical Medicine 2022;9:18-29.
43. El-Dien Mahmoud mEA, El Zahaby HMM, Moharram Ahmed Moharram AA, Asaad AFA. The Effect of Dexmedetomidine Infusion Versus Magnesium Sulphate Infusion on Emergence Delirium in Pediatric Patients Undergoing Lower Abdominal Surgery. QJM : monthly journal of the Association of Physicians 2021;114.
44. Shen S, Jiang M, Zhang H, et al. Effect of dexmedetomidine on hemodynamics and stress response of laparoscopic surgery in children. International Journal of Clinical and Experimental Medicine 2020;13:7250-7.
45. Koceroglu I, Devrim S, Bingol Tanriverdi T, Gura Celik M. The effects of dexmedetomidine and tramadol on post-operative pain and agitation, and extubation quality in paediatric patients undergoing adenotonsillectomy surgery: A randomized trial. J Clin Pharm Ther 2020;45:340-6.
46. Huang S, Luo L. Effects of sevoflurane combined with sufentanil on the outcome of children with indirect inguinal hernia. International Journal of Clinical and Experimental Medicine 2020;13:8455-62.
47. Sun Y, Liu J, Yuan X, Li Y. Effects of dexmedetomidine on emergence delirium in pediatric cardiac surgery. Minerva Pediatr 2017;69:165-73.
48. Prevent emergence delirium in paediatric patients undergoing anaesthesia by using propofol or adding an adjuvant to sevoflurane. Drugs and Therapy Perspectives 2017;33:473-7.
49. Amr S, Osman M, Ibrahim I, Zaki E. Effects of dexmedetomidine added to caudal bupivacaine on postoperative analgesia and stress response in pediatric lower abdominal surgeries. Regional Anesthesia and Pain Medicine 2014;39:e159.
50. Ibacache M, Muñoz H, Brandes V, Altermatt F. Dexmedetomidine decreases post-sevoflurane agitation in children. Revista chilena de anestesiología 2002;31:193-4.
51. Wei W, Fan Y, Liu W, et al. Combined non-intubated anaesthesia and paravertebral nerve block in comparison with intubated anaesthesia in children
52. Xu J, Zhou G, Li Y, Li N. Benefits of ultra-fast-track anesthesia for children with congenital heart disease undergoing cardiac surgery. BMC Pediatrics 2019;19.

Table S3

Table S3: General characteristics of 78 included studies.

First author / Year	Age	Operation type	N	Details of the intervention	Assessment method of EA	Timing	Route
Alansary_1 2023	2~6 y	lower abdominal or perineal surgeries	25<br>25<br>25	0.25% bupivacaine (1 ml/kg) with 1.5 µg/kg dexmedetomidine /<br>0.25% bupivacaine (1 ml/kg) with 30 µg/kg midazolam /<br>0.25% bupivacaine (1 ml/kg) with normal saline	PAED ≧ 10	after the induction of anesthesia	Perineural
Alansary_2 2023	3 months to 7 y	elective cleft lip repair	25<br>25<br>25	1 ml of bupivacaine 0.25% with 0.5 µg/kg dexmedetomidine /<br>1 ml of bupivacaine 0.25% with 2 mg/kg $MgSO_4$ /<br>1 ml of bupivacaine 0.25%.	PAED	after the induction of anesthesia	Perineural
Zhang 2022	3~7 y	tonsillectomy ± adenoidectomy	20<br>20<br>20<br>20	normal saline /<br>0.4 µg/kg dexmedetomidine /<br>0.4 µg/kg dexmedetomidine + alfentanil (10µg/kg) /<br>0.4 µg/kg dexmedetomidine + alfentanil (20µg/kg)	Aono ≥ 3	after the induction of anesthesia	intravenous
Yi 2022	5 (3.25-6.75) y<br>3 (2.25-4) y<br>median (IQR)	adenotonsillecto-my	62<br>58	1µg.kg^{-1} dexmedetomidine /<br>0.5 µg.kg^{-1} dexmedetomidine	Cole ≥ 4	after the induction of anesthesia	intravenous
Yao 2022	2~6 y	tonsillectomy ± adenoidectomy	30<br>30<br>30<br>30	no PPIA /<br>PPIA /<br>dexmedetomidine (1.0 µg/kg) /<br>dexmedetomidine (1.0 µg/kg) + PPIA	PAED	Premedication	intranasal
Verma 2022	2~8 y	all types of elective surgery	33<br>31	2 µg/kg intranasal dexmedetomidine /<br>2 µg/kg nebulized dexmedetomidine	four-point score Davis 1995	Premedication	Intranasal / nebulized
Sultana 2022	2~8 y	elective sub-umbilical surgery	60<br>60	fentanyl 1 mcg/kg /<br>dexmedetomidine 0.15 mcg/kg	PAED	after the induction of anesthesia	Intravenous
Pandey 2022	1~6 y	elective ophthalmic	38<br>38<br>38<br>38	S-saline normal saline /<br>S-Dex Dexmedetomidine 0.3 mcg/kg /<br>D-saline normal saline /<br>D-Dex Dexmedetomidine 0.3 mcg/kg	PAED ≥ 10	before the end of the surgery	Intravenous
Naveen 2022	1~4 y	ambulatory oral rehabilitation	36<br>36	dexmedetomidine 0.25 µg/kg followed by a maintenance infusion of 0.4 µ g/kg/h /<br>fentanyl 1 µg/kg over 4 min for induction, followed by a maintenance infusion of 1 µg/kg/h	Non-specified	before the end of the surgery	intravenous
Lanin D 2022	1~7 y	any surgical procedure of at least two hours duration	93<br>94<br>90	0.5 mg/kg midazolam /<br>2 µg/kg dexmedetomidine /<br>4 µg/kg dexmedetomidine	3-point scale_Jannu V 2016	Premedication	oral
Jangra 2022	5.5 ± 2.2 y<br>5.4 ± 2.0 y<br>mean ± SD	ophthalmic surgery	60<br>60	intranasal normal saline + oral 0.5 mg/kg melatonin /<br>2 µg/kg intranasal dexmedetomidine + oral plain honey placebo	PAED ≥ 10	Premedication	intranasal
Huang 2022	8~24 months	cleft palate repair	28<br>29<br>29	normal saline /<br>2 mg/kg/hour propofol /<br>0.5µg/kg/hour dexmedetomidine	PAED ≥ 10	after the induction of anesthesia	Intravenous
El-sherbiny 2022	2~8 y	strabismus surgery	40<br>40	bupivacaine 0.5% (0.08 ml/kg) alone /<br>bupivacaine 0.5% (0.08 ml/kg) + dexmedetomidine (0.5 µg/kg)	PAED ≥ 12	after the induction of anesthesia	Perineural
Ali 2022	1~8 y	cochlear implantation	25<br>25	3 µg/kg dexmedetomidine /<br>4 µg/kg dexmedetomidine	Watcha ≧ 3	Premedication	Inhalation
Zhu 2021	1~6 y	urethroplasty	40<br>40	1 ml/kg of 0.2% ropivacaine and 0.5 µg/kg dexmedetomidine /<br>1 ml/kg of 0.2% ropivacaine alone	Ramsay sedation scale (RSS) = 1	after the induction of anesthesia	Perineural
Varsha R 2021	4.5 ± 2.9 y<br>3.7 ± 2.3 y<br>mean ± SD	unilateral herniotomy	23<br>23	0.75 mL/kg of 0.25% bupivacaine and 1 µg/kg of dexmedetomidine in caudal block /<br>0.25 mL/kg of 0.25% bupivacaine with 1 µg/kg of dexmedetomidine in Ilioinguinal and iliohypogastric nerve bloc	PAED	after the induction of anesthesia	Perineural
Ming 2021	1~6 y	repair of atrioventricular septal defect (AVSD)	30<br>30<br>30	normal saline /<br>dexmedetomidine 0.2 µg/kg/h /<br>dexmedetomidine 0.4 µg/kg/h	5-point scale_Peng 2015 ≧ 2	after the induction of anesthesia	Intravenous
Elghamry 2021	3~7 y	strabismus correction surgeries	34<br>33	dexmedetomidine 0.3 µg/kg /<br>normal saline	Watcha ≧ 3	before the end of the surgery	Intravenous
Cai 2021	2~6 y	lower abdominal or perineal surgery	37<br>46<br>46	0.5 mg/kg oral midazolam and intranasal saline /<br>oral saline + 2 µg/kg intranasal dexmedetomidine /<br>0.5 mg/kg oral midazolam + 1 µg/kg intranasal dexmedetomidine	PAED ≧ 10	Premedication	Intranasal
Yao 2020	2~6 y	elective unilateral strabismus surgery	52<br>50<br>51	intranasal 2 µg/kg dexmedetomidine + oral 0.9% saline /<br>intranasal 0.9% saline + oral midazolam (0.5 mg/kg)/<br>intranasal 0.9% saline + oral 0.9% saline	PAED ≧ 10	Premedication	Intranasal
Elagamy 2020	2~5 y	adenotonsillecto-my	80<br>80	0.5 µg/kg dexmedetomidine /<br>0.1 mg/kg nalbuphine	PAED ≧ 16	after the induction of anesthesia	Intravenous
Shi 2019	2~7 y	Tonsillectomy ± adenoidectomy	45<br>45	normal saline /<br>0.5 µg/kg dexmedetomidine	PAED ≧ 10	after the induction of anesthesia	Intravenous
Bi 2019	6~48 months	Tracheobronchial foreign body removal	20<br>20	dexmedetomidine 1 µg/kg /<br>normal saline	Cole ≧ 4	Premedication	Intranasal
Abdel-Ghaffer 2019	3~6 y	Tonsillectomy ± adenoidectomy	30<br>30<br>30	Normal saline /<br>Dexmedetomidine 0.5µg/kg /<br>Dexmedetomidine 1µg/kg	Watcha ≥ 3	Premedication	Oral
Chen 2018	3~7 y	elective inguinal hernia repair	20<br>20<br>20<br>20<br>20	normal saline /<br>0.25 µg/kg dexmedetomidine /<br>0.5 µg/kg dexmedetomidine /<br>0.75 µg/kg dexmedetomidine /<br>1 µg/kg dexmedetomidine	PAED > 12	after the induction of anesthesia	Intravenous
Bhat 2018	1~8 y	inguinal hernia repair	30<br>30<br>30	normal saline /<br>0.5 µg/kg dexmedetomidine /<br>1 µg/kg dexmedetomidine	Aono ≥ 3	after the induction of anesthesia	Intravenous
Abdel-Rahman 2018	3~8 y	Strabismus surgery	30<br>30<br>30	0.5 µg /kg dexmedetomidine /<br>0.25 µg/kg dexmedetomidine /<br>normal saline	PAED > 10	before the end of the surgery	Intravenous

Table S3

Sun 2017	1~5 y	laparoscopic hernia repair	24<br>23<br>25<br>25	normal saline /<br>0.25 µg/kg dexmedetomidine /<br>0.5 µg/kg dexmedetomidine /<br>1.0 µg/kg dexmedetomidine	Cole ≧ 3	after the induction of anesthesia	Intravenous
Luo 2017	1~5 y	cleft palate repair	46<br>47	dexmedetomidine 0.5 µg/kg + sufentanil 0.2 µg/kg /<br>normal saline + fentanyl 2 µg/kg	PAED > 11	Premedication	Intravenous
Kannojia 2017	2~7 y	urogenital surgery	30<br><br>30<br>30	0.5 ml/kg bupivacaine 0.25% + 1 µg/kg dexmedetomidine /<br>0.5 ml/kg bupivacaine 0.25% + 1µg/kg fentanyl /<br>0.5 ml/kg bupivacaine 0.25% only	PAED	After the induction of anesthesia	Perineural
Govil 2017	2~8 y	cochlear implant surgery	30<br><br>30	0.5 µg/kg dexmedetomidine followed by infusion of dexmedetomidine at 0.5 µg/kg/hr /<br>100 ml normal saline over 10 mins, followed by infusion at 2 ml hr.	PAED > 16	After the induction of anesthesia	Intravenous
Ezz 2017	3~6 y	unilateral or bilateral myringotomy	45<br>45	5 mg/kg ketamine /<br>1 µg/kg dexmedetomidine	Aono ≥ 3	Premedication	Intranasal
El-Hamid 2017	3~7 y	elective tonsillectomy ± adenoidectomy	43<br>43	1 µg/kg dexmedetomidine /<br>normal saline	Aono ≥ 3	After the induction of anesthesia	Intranasal
Di 2017	3~7 y	adenotonsillecto-my	25<br>25<br>25	normal saline /<br>1 µg/kg dexmedetomidine /<br>2 µg/kg dexmedetomidine	PAED > 10	Premedication	Intravenous
Song 2016	2~6 y	outpatient elective strabismus surgery	25<br>25<br>25<br>28	normal saline /<br>dexmedetomidine 0.25 µg/kg /<br>dexmedetomidine 0.5 µg/kg /<br>dexmedetomidine 1.0 µg/kg	Aono ≥ 3	After the induction of anesthesia	Intravenous
Lin 2016	1~8 y	cataract surgeries	30<br>30<br>30	1 µg/kg dexmedetomidine /<br>2 µg/kg dexmedetomidine /<br>normal saline	PAED > 10	Premedication	intranasal
Jannu V 2016	1~7 y	elective, minor, lower abdominal surgeries	30<br>30	0.75 mg/kg midazolam /<br>4 µg/kg dexmedetomidine	3-point scale_ _Jannu V 2016	Premedication	oral
Boku 2016	10~14 months	palatoplasty	35<br>35	normal saline /<br>1 µg/kg dexmedetomidine, followed by 0.4 µg/kg/h	Cole	before the end of the surgery	Intravenous
Ali 2016	3~6 y	orthopedic surgery	30<br><br>30<br>30	ketofol (ketamine 0.25 mg/kg and propofol 1.0 mg/kg) /<br>dexmedetomidine 0.3 µg/kg /<br>normal saline	Aono ≥ 3	before the end of surgery	Intravenous
Al-Zaben 2016	1~6 y	elective lower abdominal and perineal surgeries	25<br><br>25<br><br><br>25	1 mL/kg caudal 0.25% bupivacaine + 10 mL intravenous 0.9% saline /<br>1 mL/kg caudal 0.25% bupivacaine mixed with 1 µg/kg dexmedetomidine + 10 mL intravenous 0.9% saline /<br>1 mL/kg of caudal 0.25% bupivacaine + 1 µg/kg intravenous dexmedetomidine and 10 mL intravenous 0.9% saline	Watcha ≧ 3	After the induction of anesthesia	Perineural / Intravenous
Abdelaziz 2016	1~7 y	strabismus surgeries	32<br>33<br>33	normal saline /<br>1 µg/kg dexmedetomidine /<br>0.1 mg/kg midazolam	PAED ≥ 10	Premedication	Intranasal
Yao 2015	3~7 y	elective unilateral strabismus surgery	29<br>30<br>30	normal saline /<br>dexmedetomidine 1 µg/kg /<br>dexmedetomidine 2 µg/kg	PAED ≥ 10	Premedication	Intranasal
Peng 2015	3~24 months	cleft palate repair	20<br>20	0.8 µg/kg/min dexmedetomidine /<br>normal saline	5-point scale _ Scheinin 1989 ≥ 4	After the induction of anesthesia	Intravenous
Mukherjee 2015	3~7 y	elective day care surgery	40<br>40	1 µg/kg dexmedetomidine /<br>4 µg/kg clonidine	Aono	Premedication	Intranasal
Mohamed 2015	18~38 months	elective congenital hernia surgeries	24<br><br>24	caudal epidural analgesia with 1% lidocaine 0.7 ml/kg /<br>caudal epidural analgesia with 1% lidocaine 0.7 ml/kg + dexmedetomidine 2 µg/kg.	Aono ≥ 3	After the induction of anesthesia	Perineural
Hadi 2015	3~7 y	adenotonsillecto-my	45<br><br>47	ketamine 0.15 mg/kg followed dexmedetomidine 0.3 mg/kg /<br>normal saline	PAED ≥ 10	Before the end of the surgery	Intravenous
Cho 2015	1~6 y	ambulatory unilateral orchiopexy	40<br><br>40	1.5 mL/kg of 0.15% ropivacaine with the same amount of saline compared to dexmedetomidine /<br>1.5 mL/kg of 0.15% ropivacaine with 1µg/kg dexmedetomidine	Watcha ≥ 3	After the induction of anesthesia	Perineural
Sheta 2014	3~6 y	complete dental rehabilitation	36<br>36	0.2 mg/kgmidazolam /<br>1 µg/kg dexmedetomidine	Aono ≥ 3	Premedication	Intranasal
Kim, N.Y. 2014	1~5 y	ambulatory hernioplasty or orchiopexy	20<br><br>20	dexmedetomidine 1 µg/kg bolus followed by a 0.1 µg/kg/h infusion /<br>normal saline	Watcha ≥ 3	After the induction of anesthesia	Intravenous
Bharti 2014	1~8 y	lower abdominal and perineal surgery	20<br>19<br><br>20<br><br>19	caudal block with 0.2% plain ropivacaine /<br>caudal dexmedetomidine 0.5 µg/kg, along with 0.2% plain ropivacaine /<br>caudal dexmedetomidine 1 µg/kg, along with 0.2% plain ropivacaine /<br>caudal dexmedetomidine 1.5 µg/kg, along with 0.2% plain ropivacaine	PAED ≥ 10	After the induction of anesthesia	Perineural
Abdel-Ma'boud 2014	4~6 y	inguinal hernia repair	20<br>20<br>20	1 µg/kg dexmedetomidine followed by 0.1µg/kg/hr /<br>1 mg/kg propofol /<br>normal saline infusion at the same rate of dexemeditomedine	Watcha ≥ 3	After the induction of anesthesia	Intravenous
He 2013	3~7 y	elective minor surface surgery	26<br>29<br>32	Normal saline /<br>0.5 µg/kg dexmedetomidine /<br>1 µg/kg dexmedetomidine /	Cole ≧ 4	After the induction of anesthesia	Intravenous
El-Rahmawy 2013	2~6 y	surgery for fracture femur	14<br>14	1 mg/kg bupivacaine 0.25% /<br>1 mg/kg bupivacaine 0.25% with dexmedetomidine 2 µg/kg	Watcha ≧ 3	After the induction of anesthesia	Perineural
Chen 2013	2~7 y	elective strabismus surgery	24<br>27<br><br>27	normal saline /<br>dexmedetomidine 1µg/kg, followed by infusion at 1 µg/kg/hr /<br>ketamine 1 mg/kg, followed by infusion at 1 mg/kg/hr	PAED ≥ 10	After the induction of anesthesia	Intravenous

Table S3

Ali 2013	2~6 y	adenotonsillecto-<br>my	40<br>40<br>40	Normal saline /<br>Propofol 1 mg/kg<br>Dexmedetomidine 0.3 μg/kg	Aono ≥3	Before the end of the surgery	Intravenous
Lili 2012	3~7 y	vitreoretinal surgery	30<br>30	dexmedetomidine 0.5 μg/kg /<br>normal saline	Aono ≥ 3	After the induction of anesthesia	Intravenous
Anand 2011	6 months~6 y	lower abdominal surgeries	30<br>30	0.25% ropivacaine 1 ml/kg + 0.5 ml normal saline /<br>0.25% ropivacaine 1 ml/kg with dexmedetomidine 2 μg/kg	Aono	After the induction of anesthesia	Perineural
Sato 2010	2.9 ± 2.5 y<br>3.6 ± 2.2 y<br>mean ± SD	Ambulatory surgery	42<br>39	normal saline /<br>dexmedetomidine 0.3 μg/kg	Aono ≥ 3	After the induction of anesthesia	Intravenous
Patel 2010	3.8 ± 1.5 y<br>4.2 ± 2.1 y<br>mean ± SD	elective tonsillectomy ± adenoidectomy	61<br>61	Fentanyl 1μg/kg /<br>Dexmedetomidine 2μg/kg, followed by 0.7 μg/kg/h	PAED ≧ 10	After the induction of anesthesia	Intranasal
Saadawy 2009	1~6 y	unilateral inguinal hernia/orchidopex-<br>y	30<br>30	bupivacaine 2.5 mg/kg /<br>bupivacaine 2.5 mg/kg + dexmedetomidine 1μg/kg	Aono ≥ 3	After the induction of anesthesia	Perineural
Erdil 2009	2~7 y	adenoidectomy with or without bilateral myringotomy and insertion of tubes	30<br>30<br>30	normal saline /<br>fentanyl 2.5 μg/kg /<br>dexmedetomidine 0.5 μg/kg	Cole ≥ 4	After the induction of anesthesia	Intravenous
Isik 2006	53.1 ± 24.4 months<br>49.8 ± 31.4 months<br>mean ± SD	cranial MRI scanning	21<br>21	dexmedetomidine 1μg/kg /<br>normal saline	Cole ≧ 4	After the induction of anesthesia	Intravenous
Shukry 2005	59 ± 25.4 months<br>48 ± 33.4 months<br>mean ± SD	elective outpatient surgical procedure	23<br>23	dexmedetomidine 0.2μg/kg/h /<br>normal saline	Watcha ≥ 3	After the induction of anesthesia	Intravenous
Guler 2005	3~7 y	adenotonsillectom-<br>y	30<br>30	dexmedetomidine 0.5 μg/kg /<br>normal saline	Cole ≧ 4	Before the end of the surgery	Intravenous
Ibacache 2004	3.8 ± 1.5 y<br>4.4 ± 1.7 y<br>4.4 ± 1.9 y<br>mean ± SD	inguinal hernia repair, orchiopexy, or circumcision	30<br>30<br>30	normal saline /<br>dexmedetomidine 0.15 μg/kg /<br>dexmedetomidine 0.3 μg/kg	Aono ≥ 3	After the induction of anesthesia	Intravenous
Pestieau 2011	6 months~6 y	elective bilateral myringotomy	27<br>23<br>23<br>28	normal saline /<br>fentanyl 2μg/kg /<br>dexmedetomidine 1μg/kg /<br>dexmedetomidine 2μg/kg	Watcha ≧ 2	After the induction of anesthesia	Intranasal
Abdel-Ghaffar 2018	3~7 y	bone marrow aspiration and biopsy	30<br>30<br>30	0.2 mg/kg midazolam /<br>2 μg/kg dexmedetomidine /<br>2 mg/kg ketamine	3-point scale _ Abdel-Ghaffar 2018 ≥ 2	Premedication	Inhalation
Hauber 2015	6.1 ± 1.6 y<br>5.8 ± 1.6 y<br>mean ± SD	tonsillectomy ± adenoidectomy, ± myringotomy, ± tympanostomy tube insertion	19<br>5<br>19<br>8	0.5 μg/kg dexmedetomidine /<br>normal saline	PAED > 10	Before the end of the surgery	Intravenous
Lundblad 2015	18 months ~ 8 y	outpatient inguinal hernia surgery	21<br>22	ropivacaine 0.197% /<br>ropivacaine 0.197% + dexmedetomidine 0.3 μg/kg	PAED ≥ 11	After the induction of anesthesia	Perineural
Makkar 2016	2~8 y	elective infra-umbilical surgery	32<br>36<br>32	0.3 μg/kg dexmedetomidine /<br>1 mg/kg propofol /<br>normal saline	PAED ≥ 10	Before the end of the surgery	Intravenous
Mountain 2011	1~6 y	dental restoration and possible tooth extraction	22<br>19	4 μg/kg dexmedetomidine /<br>0.5 mg/kg midazolam	PAED ≥ 10	Premedication	Oral
Wang 2020	3~6 y	full-mouth dental rehabilitation	30<br>30	0.5 mg/kg midazolam (oral) /<br>2 μg/kg dexmedetomidine (intranasal)	PAED ≥ 10	Premedication	Intranasal
Yao 2018	2~5 y	unilateral inguinal hernia repair	30<br>30<br>30	caudal 0.25% levobupivacaine 1mL/kg + dexmedetomidine 1 μg/kg /<br>caudal 0.25% levobupivacaine 1 mL/kg + IV dexmedetomidine 1 μg/kg /<br>caudal 0.25% levobupivacaine 1 mL/kg	PAED ≥ 12	After the induction of anesthesia	Perineural / intravenous
Zhang 2020	3.0 [2~4] y<br>2.0 [1~4] y<br>median [IQR]	elective interventional cardiac catheterisation	67<br>67	1.5 μg/kg dexmedetomidine /<br>normal saline	Aono	Premedication	Intranasal
Bong 2015	2~7 y	MRI under general anaesthesia	40<br>39<br>41	0.3 μg/kg dexmedetomidine /<br>1 mg/kg propofol /<br>normal saline	PAED ≥ 10	After the induction of anesthesia	Intravenous
Surana 2017	14.5 [9~31.5] months<br>22 [10~42.75] months<br>median [IQR]	cleft palate surgery	30<br>30	0.05 mg/kg midazolam followed by normal saline at 0.5 ml/kg/h /<br>1 μg/kg dexmedetomidine followed by a maintenance infusion of 0.5 μg/kg/h	Watcha	After the induction of anesthesia	Intravenous
Hamawy 2019	5.34 ± 1.65 y<br>6.08 ± 1.73 y<br>5.76 ± 2.0 y<br>mean ± SD	unilateral strabismus correction surgery	25<br>25<br>25	1 μg/kg dexmedetomidine /<br>peribulbar injection of 2–5 ml of local anesthetic in the form of lidocaine: bupivacaine (1:1 ratio) /<br>placebo (Group C received only general anesthesia)	PAED ≥10	Premedication	Intranasal
Liang 2022	1~5 y	Under general anesthesia	34<br>34	1 μg/kg dexmedetomidine /<br>normal saline	restlessness score (RS) ≧ 3	Premedication	Intranasal

Data presented as mean ± 1 SD (standard deviation) or median ± IQR (interquartile range)

Table S4

Table S4. TSA for subgroup analysis of EA incidence.

Subgroups	RRR% (MD)	IIA% (variance)	ICA%	D^2%	Required IS	Reach IS	Cross TSMB	Cross FB	Evidence
**Dichotomous outcomes**									
Incidence of EA									
Dex vs Placebo	30	33.88	48.4	56	827	Yes	Yes	No	Firm
Different administration route									
Intravenous	30	38.15	54.5	75	1190	Yes	Yes	No	Firm
Perineural	30	21.98	31.4	0	693	Yes	Yes	No	Firm
Intranasal	30	31.15	44.5	38	671	Yes	Yes	No	Firm
Oral	30	16.31	23.3	0	1021	No	No	No	Absent

TSA, trial sequential analysis; EA, emergence agitation; RRR, relative risk reduction; IIA, the incidence in the intervention arm; ICA, the incidence in the control arm; D^2, diversity; IS, information size; TSMB, trial sequential monitoring boundary; FB, futility boundary; MD, mean difference. Error α and 1-β were defined as 5% and 80%, respectively, in each model; For dichotomous data, RRR was defined as 30%, ICA was calculated from the average incidence in the control group, D^2 was set as model variance-based

Table S5

Table S5. Results of subgroup analysis by different routes of Dex administration, including intravenous, perineural, intranasal, and oral. RR, risk ratio.

Subgroups	No. of studies / participants	Heterogeneity / model of pool	Effect size (95% CI)	P-value	Subgroup difference
**Different administration routes**					P=0.23
Intravenous	33/4296	I^2=62% / Random effect	RR: 0.31 [0.25-0.40]	P<.00001	
Perineural	12/707	I^2=0% / Fixed effect	RR: 0.22 [0.14-0.33]	P<.00001	
Intranasal	11/886	I^2=34% / Fixed effect	RR: 0.31 [0.24-0.40]	P<.00001	
Oral	1/90	I^2= not applicable	RR: 0.71 [0.30-1.69]	P=0.44	

Table S6

Table S6. Results of subgroup analysis by sample sizes. RR, risk ratio.

Subgroup	No. of studies / participants	Heterogeneity / model of pool	Effect size (95% CI)	P-value	Subgroup difference
**Different sample sizes**					
≦60	23/ 1135	I^2=0% / Fixed effect	RR : 0.25 [0.20-0.32]	P<.00001	P=0.22
> 60	33/ 3102	I^2=69% / Random effect	RR : 0.35 [0.28-0.44]	P<.00001	

Figure S1

Study or Subgroup	Dex Events	Dex Total	Placebo Events	Placebo Total	Weight	Risk Ratio M-H, Fixed, 95% CI
**2.2.1 ≤60**						
Abdel-Ma'boud 2014	3	20	12	20	1.3%	0.25 [0.08, 0.75]
Alansary_1 2023	0	25	5	25	0.6%	0.09 [0.01, 1.56]
Alansary_2 2023	4	25	7	25	0.7%	0.57 [0.19, 1.71]
Ali 2016	5	30	27	30	2.8%	0.19 [0.08, 0.42]
Al-Zaben 2016	0	25	8	25	0.9%	0.06 [0.00, 0.97]
Bi 2019	5	20	14	20	1.5%	0.36 [0.16, 0.80]
Chen 2013	3	27	11	24	1.2%	0.24 [0.08, 0.77]
El-Rahmawy 2013	1	14	8	14	0.8%	0.13 [0.02, 0.87]
Erdil 2009	5	30	14	30	1.5%	0.36 [0.15, 0.87]
Govil 2017	1	30	12	30	1.3%	0.08 [0.01, 0.60]
Guler 2005	5	30	17	30	1.8%	0.29 [0.12, 0.69]
Hamawy 2019	8	25	19	25	2.0%	0.42 [0.23, 0.78]
Huang 2022	6	29	24	28	2.6%	0.24 [0.12, 0.50]
Isik 2006	0	21	7	21	0.8%	0.07 [0.00, 1.10]
Kim, N.Y. 2014	1	20	11	20	1.1%	0.09 [0.01, 0.64]
Lili 2012	3	30	13	30	1.4%	0.23 [0.07, 0.73]
Lundblad 2015	0	22	4	21	0.5%	0.11 [0.01, 1.86]
Mohamed 2015	2	24	11	24	1.1%	0.18 [0.04, 0.73]
Peng 2015	3	20	18	20	1.9%	0.17 [0.06, 0.48]
Saadawy 2009	2	30	8	30	0.8%	0.25 [0.06, 1.08]
Shukry 2005	6	23	14	23	1.5%	0.43 [0.20, 0.92]
Yao 2018	2	30	8	30	0.8%	0.25 [0.06, 1.08]
Zhang 2022	5	20	10	20	1.0%	0.50 [0.21, 1.20]
**Subtotal (95% CI)**		**570**		**565**	**29.8%**	**0.25 [0.20, 0.32]**
Total events	70		282			
Heterogeneity: Chi^2 = 17.42, df = 22 (P = 0.74); I^2 = 0%						
Test for overall effect: Z = 12.00 (P < 0.00001)						

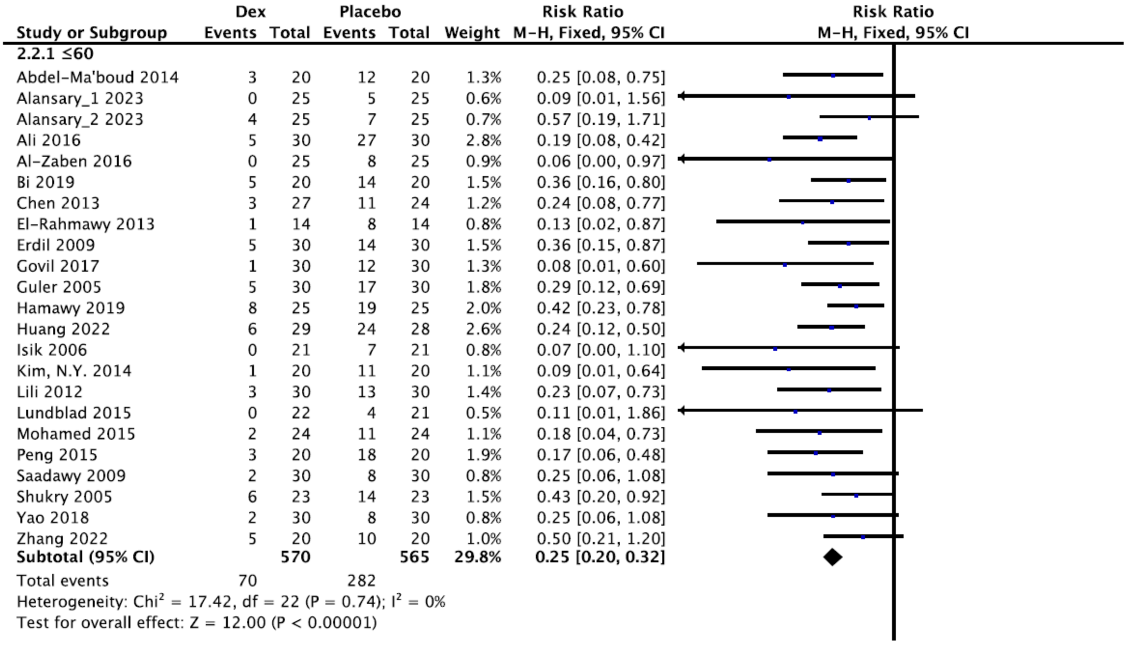

Study or Subgroup	Dex Events	Dex Total	Placebo Events	Placebo Total	Weight	Risk Ratio M-H, Random, 95% CI
**2.2.2 >60**						
Abdelaziz 2016	4	33	15	32	1.8%	0.26 [0.10, 0.70]
Abdel-Ghaffer 2019	10	60	7	30	2.1%	0.71 [0.30, 1.69]
Abdel-Rahman 2018	5	60	10	30	1.9%	0.25 [0.09, 0.67]
Ali 2013	5	40	22	40	2.1%	0.23 [0.10, 0.54]
Bharti 2014	0	58	4	20	0.4%	0.04 [0.00, 0.70]
Bhat 2018	6	60	13	30	2.1%	0.23 [0.10, 0.55]
Bong 2015	17	40	13	41	2.8%	1.34 [0.75, 2.38]
Cai 2021	7	46	10	37	2.1%	0.56 [0.24, 1.34]
Chen 2013	3	27	11	24	0.0%	0.15 [0.03, 0.63]
Chen 2018	2	80	6	20	1.1%	0.08 [0.02, 0.38]
Cho 2015	3	40	18	40	1.6%	0.17 [0.05, 0.52]
Di 2017	0	50	6	25	0.4%	0.04 [0.00, 0.67]
Elghamry 2021	6	34	19	33	2.3%	0.31 [0.14, 0.67]
El-Hamid 2017	3	43	25	43	1.6%	0.12 [0.04, 0.37]
El-sherbiny 2022	7	40	15	40	2.3%	0.47 [0.21, 1.02]
Hauber 2015	69	195	125	198	3.7%	0.56 [0.45, 0.70]
He 2013	17	61	11	26	2.7%	0.66 [0.36, 1.20]
Ibacache 2004	8	60	11	30	2.2%	0.36 [0.16, 0.81]
Liang 2022	2	34	6	34	1.1%	0.33 [0.07, 1.54]
Lin 2016	10	60	24	30	2.8%	0.21 [0.12, 0.38]
Luo 2017	3	46	38	47	1.6%	0.08 [0.03, 0.24]
Makkar 2016	3	32	13	32	1.5%	0.23 [0.07, 0.73]
Ming 2021	29	60	20	30	3.4%	0.72 [0.50, 1.04]
Pandey 2022	4	38	30	38	1.9%	0.13 [0.05, 0.34]
Pestieau 2011	14	51	11	27	2.7%	0.67 [0.36, 1.27]
Sato 2010	11	39	27	42	2.9%	0.44 [0.25, 0.76]
Shi 2019	14	45	24	45	3.0%	0.58 [0.35, 0.97]
Song 2016	29	78	15	25	3.2%	0.62 [0.40, 0.95]
Sun 2017	11	73	11	24	2.5%	0.33 [0.16, 0.66]
Yao 2015	6	60	14	29	2.1%	0.21 [0.09, 0.48]
Yao 2020	6	52	25	51	2.2%	0.24 [0.11, 0.53]
Zhang 2020	6	67	13	67	2.0%	0.46 [0.19, 1.14]
Zhu 2021	1	40	9	40	0.7%	0.11 [0.01, 0.84]
**Subtotal (95% CI)**		**1775**		**1276**	**66.7%**	**0.35 [0.28, 0.44]**
Total events	318		610			
Heterogeneity: Tau^2 = 0.26; Chi^2 = 98.85, df = 31 (P < 0.00001); I^2 = 69%						
Test for overall effect: Z = 8.77 (P < 0.00001)						
**Total (95% CI)**		**2345**		**1841**	**100.0%**	**0.32 [0.27, 0.38]**
Total events	388		892			
Heterogeneity: Tau^2 = 0.23; Chi^2 = 132.41, df = 54 (P < 0.00001); I^2 = 59%						
Test for overall effect: Z = 12.13 (P < 0.00001)						
Test for subgroup differences: Chi^2 = 1.48, df = 1 (P = 0.22), I^2 = 32.6%						

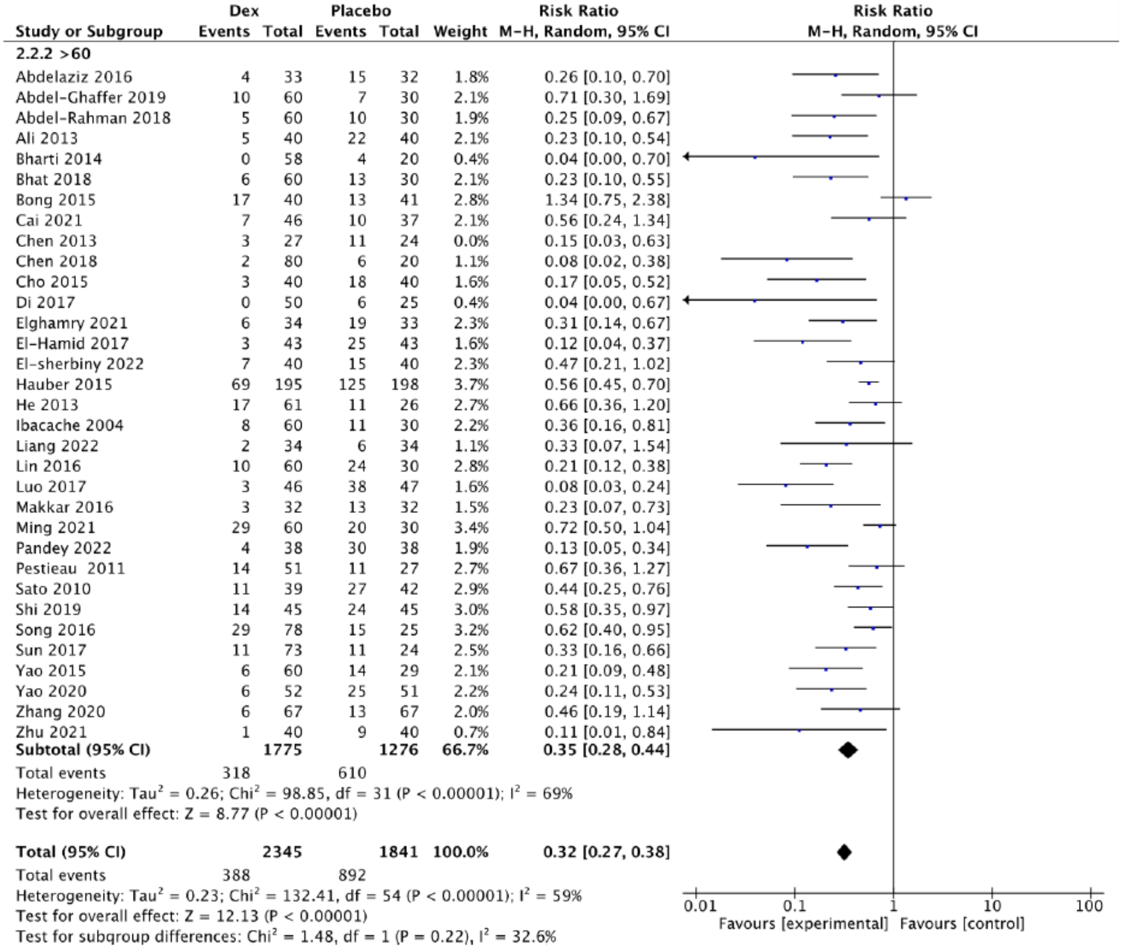

Figure S1. Risk of EA in children with or without Dex after Sevo exposure analyzed with different sample sizes. Forest plot for EA incidence by sample size (≦60 or > 60): Dex vs. Control (Placebo).

Figure S2

Study or Subgroup	Dex Events	Dex Total	Control Events	Control Total	Weight	Risk Ratio M-H, Random, 95% CI
Abdelaziz 2016	4	33	15	32	1.9%	0.26 [0.10, 0.70]
Abdel-Ghaffer 2019	10	60	7	30	2.2%	0.71 [0.30, 1.69]
Abdel-Ma'boud 2014	3	20	12	20	1.7%	0.25 [0.08, 0.75]
Abdel-Rahman 2018	5	60	10	30	1.9%	0.25 [0.09, 0.67]
Alansary_1 2023	0	25	5	25	0.4%	0.09 [0.01, 1.56]
Alansary_2 2023	4	25	7	25	1.7%	0.57 [0.19, 1.71]
Ali 2013	5	40	22	40	2.2%	0.23 [0.10, 0.54]
Ali 2016	5	30	27	30	2.3%	0.19 [0.08, 0.42]
Al-Zaben 2016	0	25	8	25	0.4%	0.06 [0.00, 0.97]
Bharti 2014	0	58	4	20	0.4%	0.04 [0.00, 0.70]
Bhat 2018	6	60	13	30	2.2%	0.23 [0.10, 0.55]
Bi 2019	5	20	14	20	2.3%	0.36 [0.16, 0.80]
Bong 2015	17	40	13	41	2.9%	1.34 [0.75, 2.38]
Cai 2021	7	46	10	37	0.0%	0.56 [0.24, 1.34]
Chen 2013	3	27	11	24	0.0%	0.24 [0.08, 0.77]
Chen 2018	2	80	6	20	1.1%	0.08 [0.02, 0.38]
Cho 2015	3	40	18	40	1.6%	0.17 [0.05, 0.52]
Di 2017	0	50	6	25	0.4%	0.04 [0.00, 0.67]
Elghamry 2021	6	34	19	33	2.4%	0.31 [0.14, 0.67]
El-Hamid 2017	3	43	25	43	1.7%	0.12 [0.04, 0.37]
El-Rahmawy 2013	1	14	8	14	0.8%	0.13 [0.02, 0.87]
El-sherbiny 2022	7	40	15	40	2.4%	0.47 [0.21, 1.02]
Erdil 2009	5	30	14	30	2.1%	0.36 [0.15, 0.87]
Govil 2017	1	30	12	30	0.7%	0.08 [0.01, 0.60]
Guler 2005	5	30	17	30	2.2%	0.29 [0.12, 0.69]
Hamawy 2019	8	25	19	25	2.8%	0.42 [0.23, 0.78]
Hauber 2015	69	195	125	198	3.8%	0.56 [0.45, 0.70]
He 2013	17	61	11	26	2.8%	0.66 [0.36, 1.20]
Huang 2022	6	29	24	28	2.5%	0.24 [0.12, 0.50]
Ibacache 2004	8	60	11	30	2.3%	0.36 [0.16, 0.81]
Isik 2006	0	21	7	21	0.4%	0.07 [0.00, 1.10]
Kim, N.Y. 2014	1	20	11	20	0.8%	0.09 [0.01, 0.64]
Liang 2022	2	34	6	34	1.1%	0.33 [0.07, 1.54]
Lili 2012	3	30	13	30	1.6%	0.23 [0.07, 0.73]
Lin 2016	10	60	24	30	2.9%	0.21 [0.12, 0.38]
Lundblad 2015	0	22	4	21	0.4%	0.11 [0.01, 1.86]
Luo 2017	3	46	38	47	1.7%	0.08 [0.03, 0.24]
Makkar 2016	3	32	13	32	1.6%	0.23 [0.07, 0.73]
Ming 2021	29	60	20	30	3.5%	0.72 [0.50, 1.04]
Mohamed 2015	2	24	11	24	1.3%	0.18 [0.04, 0.73]
Pandey 2022	4	38	30	38	2.0%	0.13 [0.05, 0.34]
Peng 2015	3	20	18	20	1.8%	0.17 [0.06, 0.48]
Pestieau 2011	14	51	11	27	2.7%	0.67 [0.36, 1.27]
Saadawy 2009	2	30	8	30	1.2%	0.25 [0.06, 1.08]
Sato 2010	11	39	27	42	3.0%	0.44 [0.25, 0.76]
Shi 2019	14	45	24	45	3.1%	0.58 [0.35, 0.97]
Shukry 2005	6	23	14	23	2.4%	0.43 [0.20, 0.92]
Song 2016	29	78	15	25	3.3%	0.62 [0.40, 0.95]
Sun 2017	11	73	11	24	2.6%	0.33 [0.16, 0.66]
Yao 2015	6	60	14	29	2.2%	0.21 [0.09, 0.48]
Yao 2018	2	30	8	30	1.2%	0.25 [0.06, 1.08]
Yao 2020	6	52	25	51	2.3%	0.24 [0.11, 0.53]
Zhang 2020	6	67	13	67	2.1%	0.46 [0.19, 1.14]
Zhang 2022	5	20	10	20	2.1%	0.50 [0.21, 1.20]
Zhu 2021	1	40	9	40	0.7%	0.11 [0.01, 0.84]
**Total (95% CI)**		**2272**		**1780**	**100.0%**	**0.32 [0.26, 0.38]**
Total events	378		871			

Heterogeneity: $Tau^2 = 0.24$; $Chi^2 = 131.30$, df = 52 ($P < 0.00001$); $I^2 = 60\%$

Test for overall effect: Z = 11.90 ($P < 0.00001$)

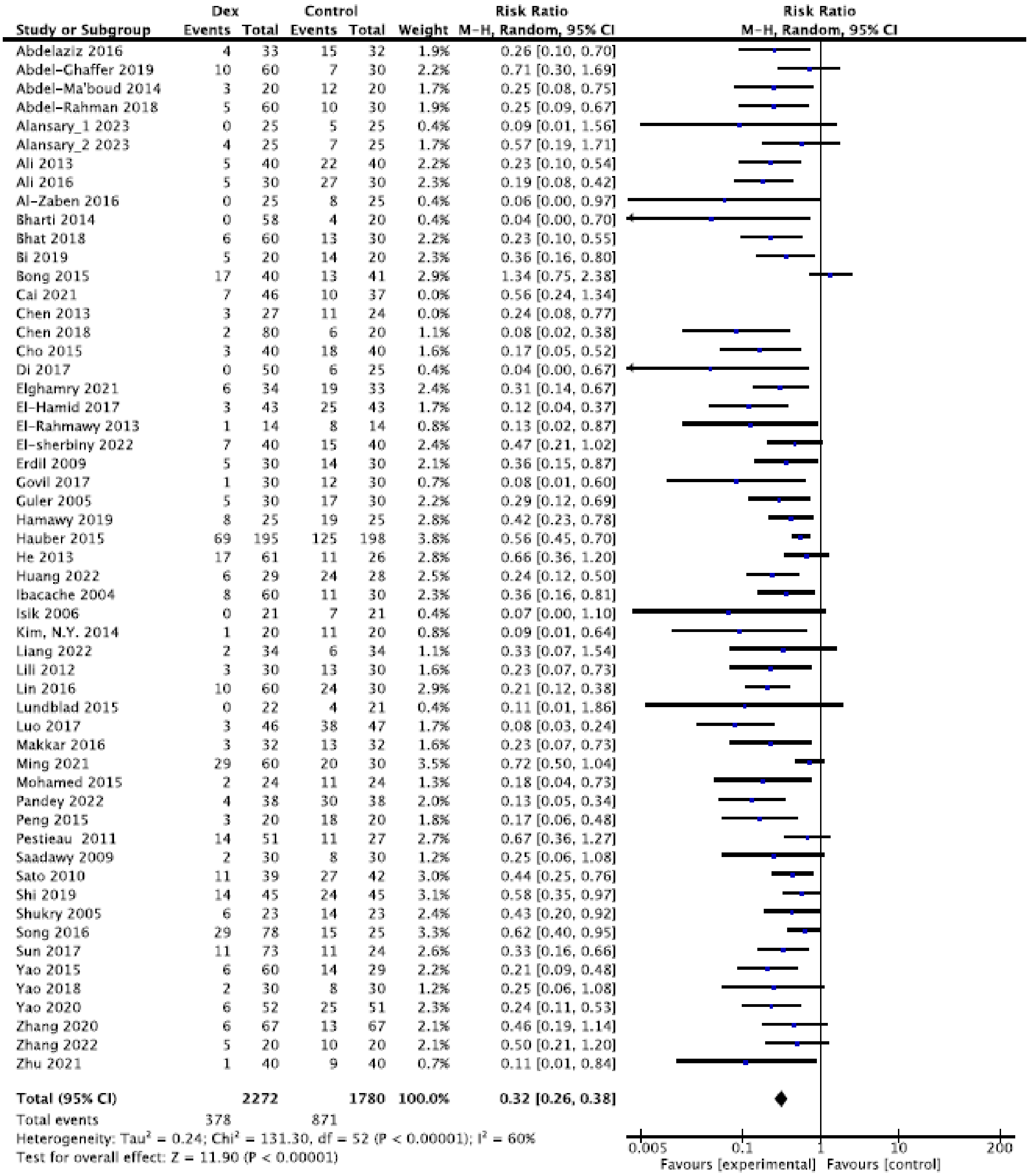

Figure S2. Risk of EA in children with or without Dex after Sevo exposure, with exclusion of high risk-of-bias studies. Forest plot for EA incidence: Dex vs. Control (Placebo).

Figure S3

(a)

Study or Subgroup	Dex Events	Dex Total	Placebo Events	Placebo Total	Weight	Risk Ratio M-H, Random, 95% CI
**2.1.1 Intravenous**						
Abdel-Ma'boud 2014	3	20	12	20	1.6%	0.25 [0.08, 0.75]
Abdel-Rahman 2018	5	60	10	30	1.8%	0.25 [0.09, 0.67]
Ali 2013	5	40	22	40	2.1%	0.23 [0.10, 0.54]
Ali 2016	5	30	27	30	2.2%	0.19 [0.08, 0.42]
Al-Zaben 2016	2	25	8	25	1.1%	0.25 [0.06, 1.06]
Bhat 2018	6	60	13	30	2.1%	0.23 [0.10, 0.55]
Bong 2015	17	40	13	41	2.8%	1.34 [0.75, 2.38]
Chen 2013	3	27	11	24	1.5%	0.24 [0.08, 0.77]
Chen 2018	2	80	6	20	1.0%	0.08 [0.02, 0.38]
Di 2017	0	50	6	25	0.4%	0.04 [0.00, 0.67]
Elghamry 2021	6	34	19	33	2.2%	0.31 [0.14, 0.67]
Erdil 2009	5	30	14	30	2.0%	0.36 [0.15, 0.87]
Govil 2017	1	30	12	30	0.7%	0.08 [0.01, 0.60]
Guler 2005	5	30	17	30	2.1%	0.29 [0.12, 0.69]
Hauber 2015	69	195	125	198	3.7%	0.56 [0.45, 0.70]
He 2013	7	61	11	26	2.1%	0.27 [0.12, 0.62]
Huang 2022	6	29	24	28	2.4%	0.24 [0.12, 0.50]
Ibacache 2004	8	60	11	30	2.2%	0.36 [0.16, 0.81]
Isik 2006	0	21	7	21	0.4%	0.07 [0.00, 1.10]
Kim, N.Y. 2014	1	20	11	20	0.7%	0.09 [0.01, 0.64]
Lili 2012	3	30	13	30	1.5%	0.23 [0.07, 0.73]
Luo 2017	3	46	38	47	1.6%	0.08 [0.03, 0.24]
Makkar 2016	3	32	13	32	1.5%	0.23 [0.07, 0.73]
Ming 2021	29	60	20	30	3.4%	0.72 [0.50, 1.04]
Pandey 2022	4	38	30	38	1.9%	0.13 [0.05, 0.34]
Peng 2015	3	20	18	20	1.7%	0.17 [0.06, 0.48]
Sato 2010	11	39	27	42	2.9%	0.44 [0.25, 0.76]
Shi 2019	14	45	24	45	3.0%	0.58 [0.35, 0.97]
Shukry 2005	6	23	14	23	2.3%	0.43 [0.20, 0.92]
Song 2016	29	78	15	25	3.2%	0.62 [0.40, 0.95]
Sun 2017	11	73	11	24	2.5%	0.33 [0.16, 0.66]
Yao 2018	2	30	8	30	1.1%	0.25 [0.06, 1.08]
Zhang 2022	5	20	10	20	2.0%	0.50 [0.21, 1.20]
**Subtotal (95% CI)**		**1476**		**1137**	**63.4%**	**0.31 [0.25, 0.40]**
Total events	279		620			
Heterogeneity: Tau² = 0.26; Chi² = 94.44, df = 32 (P < 0.00001); I² = 66%						
Test for overall effect: Z = 9.57 (P < 0.00001)						
**2.1.2 Perineural**						
Alansary_1 2023	0	25	5	25	0.4%	0.09 [0.01, 1.56]
Alansary_2 2023	4	25	7	25	1.6%	0.57 [0.19, 1.71]
Al-Zaben 2016	0	25	8	25	0.4%	0.06 [0.00, 0.97]
Bharti 2014	0	58	4	20	0.4%	0.04 [0.00, 0.70]
Cho 2015	3	40	18	40	1.5%	0.17 [0.05, 0.52]
El-Rahmawy 2013	1	14	8	14	0.7%	0.13 [0.02, 0.87]
El-sherbiny 2022	7	40	15	40	2.2%	0.47 [0.21, 1.02]
Lundblad 2015	0	22	4	21	0.4%	0.11 [0.01, 1.86]
Mohamed 2015	2	24	11	24	1.2%	0.18 [0.04, 0.73]
Saadawy 2009	2	30	8	30	1.1%	0.25 [0.06, 1.08]
Yao 2018	2	30	8	30	1.1%	0.25 [0.06, 1.08]
Zhu 2021	1	40	9	40	0.7%	0.11 [0.01, 0.84]
**Subtotal (95% CI)**		**373**		**334**	**11.6%**	**0.26 [0.17, 0.39]**
Total events	22		105			
Heterogeneity: Tau² = 0.00; Chi² = 10.50, df = 11 (P = 0.49); I² = 0%						
Test for overall effect: Z = 6.30 (P < 0.00001)						
**2.1.3 Intranasal**						
Abdelaziz 2016	4	33	15	32	1.8%	0.26 [0.10, 0.70]
Bi 2019	5	20	14	20	2.2%	0.36 [0.16, 0.80]
Cai 2021	7	46	10	37	2.1%	0.56 [0.24, 1.34]
El-Hamid 2017	3	43	25	43	1.6%	0.12 [0.04, 0.37]
Hamawy 2019	8	25	19	25	2.7%	0.42 [0.23, 0.78]
Liang 2022	2	34	6	34	1.0%	0.33 [0.07, 1.54]
Lin 2016	10	60	24	30	2.7%	0.21 [0.12, 0.38]
Pestieau 2011	14	51	11	27	2.6%	0.67 [0.36, 1.27]
Yao 2015	6	60	14	29	2.1%	0.21 [0.09, 0.48]
Yao 2020	6	52	25	51	2.2%	0.24 [0.11, 0.53]
Zhang 2020	6	67	13	67	2.0%	0.46 [0.19, 1.14]
**Subtotal (95% CI)**		**491**		**395**	**22.9%**	**0.33 [0.24, 0.44]**
Total events	71		176			
Heterogeneity: Tau² = 0.09; Chi² = 15.25, df = 10 (P = 0.12); I² = 34%						
Test for overall effect: Z = 7.16 (P < 0.00001)						

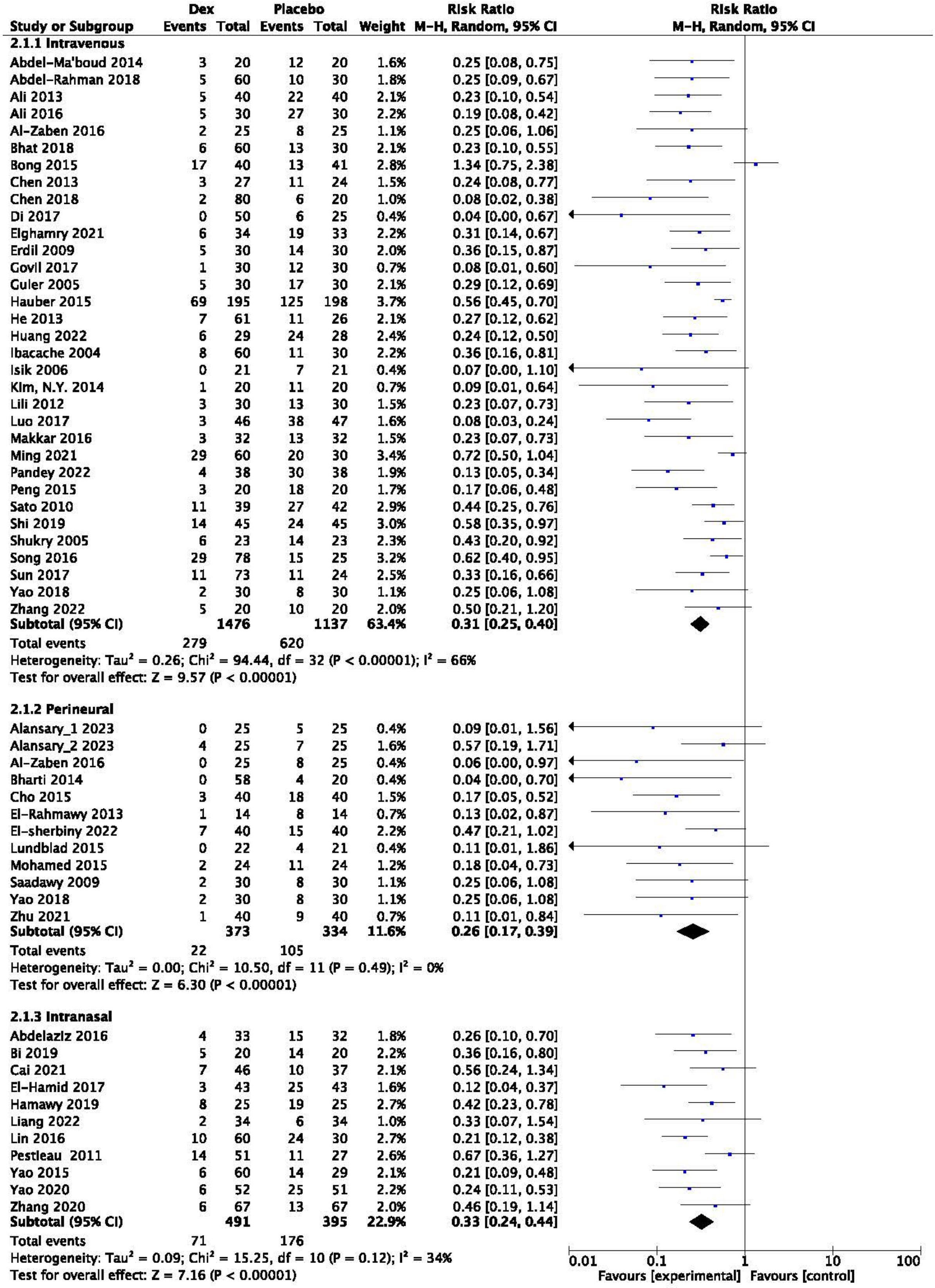

Figure S3

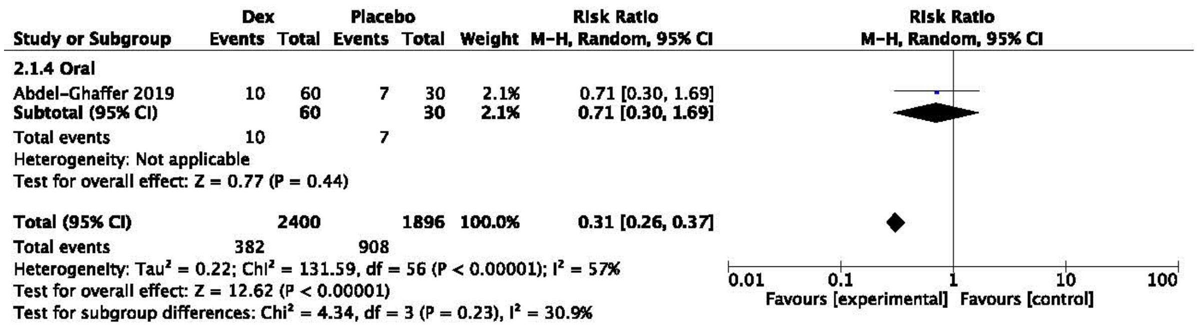

(b)

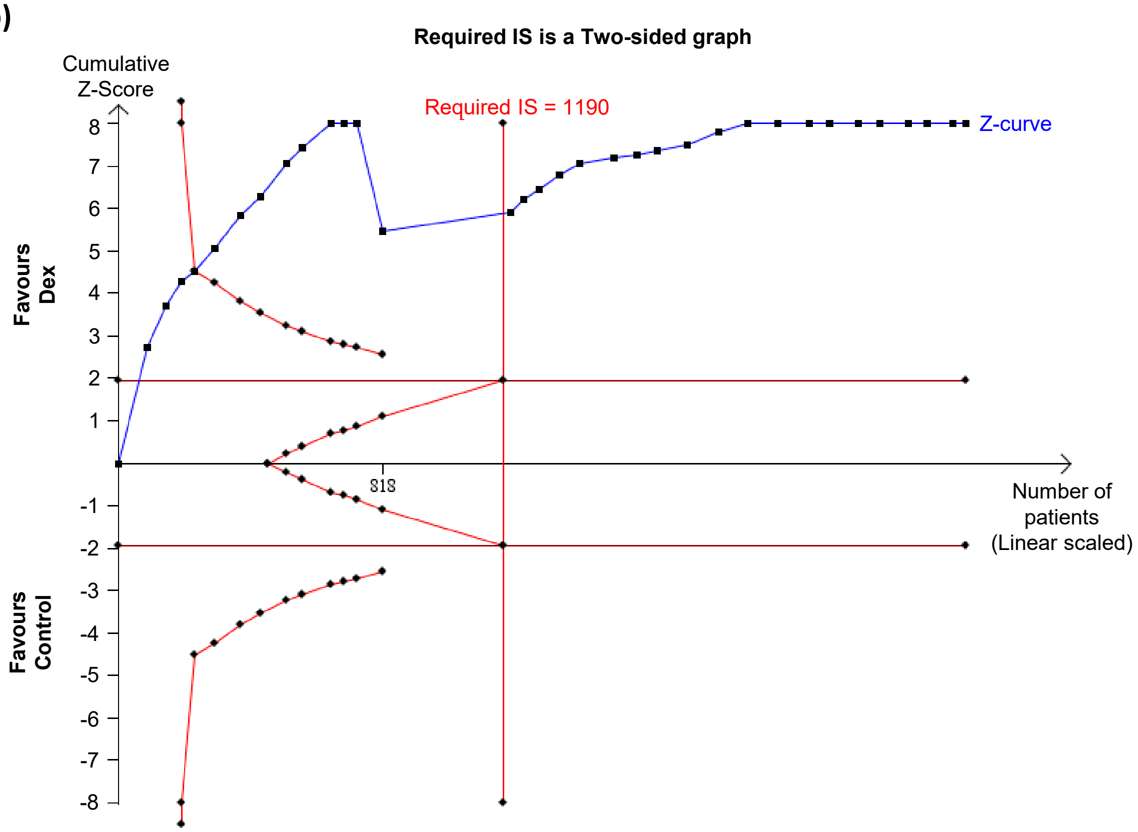

Figure S3. Risk of EA in children with or without Dex using different routes of administration after Sevo exposure. (a) Forest plot for EA incidence by different administration route: Dex vs. Control (Placebo). (b) TSA for EA incidence in intravenous (IV) route: Dex vs. Control (Placebo). Required IS, required information size.

Figure S4

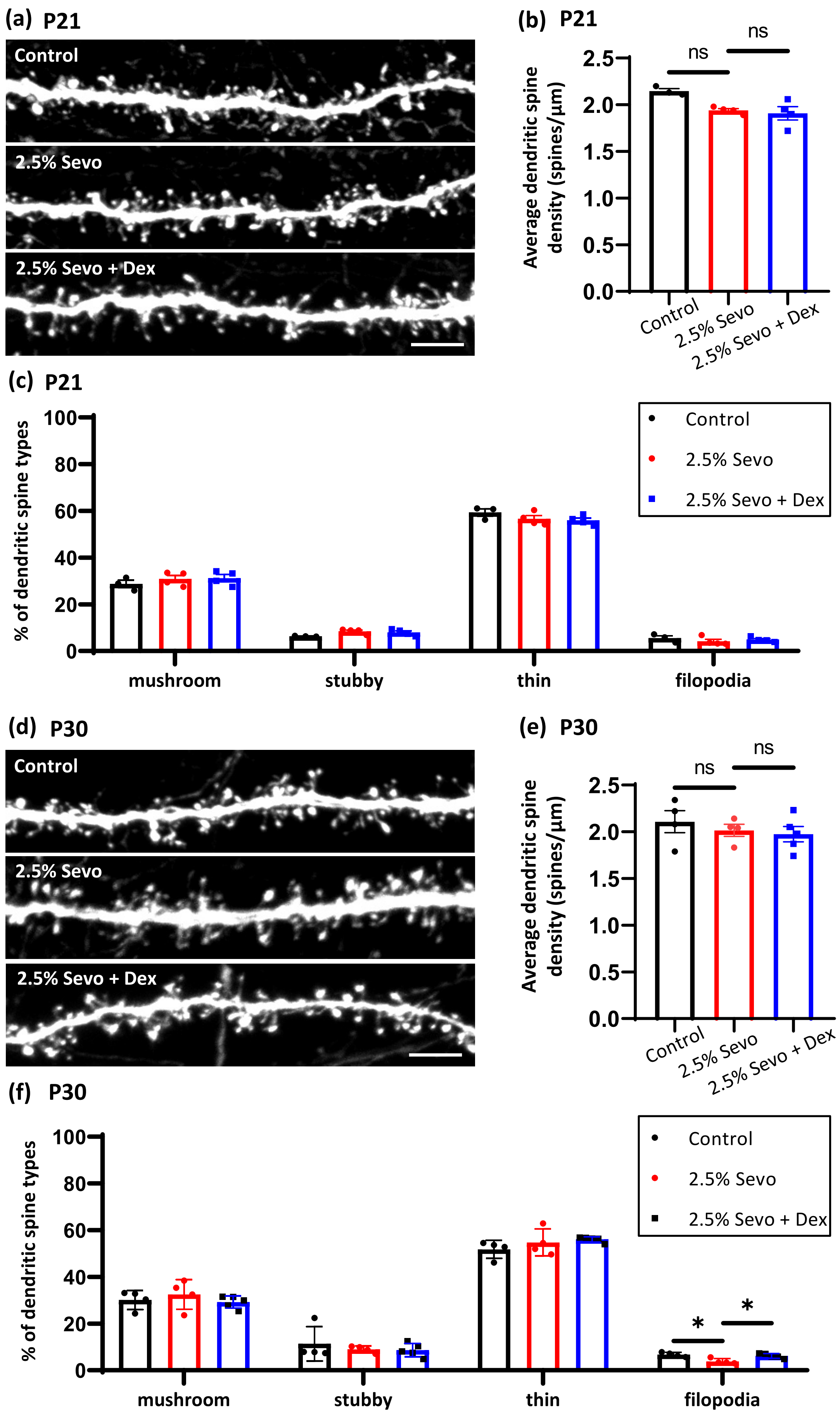

(a) P21
Control
2.5% Sevo
2.5% Sevo + Dex
(b) P21
Average dendritic spine density (spines/μm)
ns
ns
2.5
2.0
1.5
1.0
0.5
0.0
Control
2.5% Sevo
2.5% Sevo + Dex
(c) P21
% of dendritic spine types
100
80
60
40
20
0
Control
2.5% Sevo
2.5% Sevo + Dex
mushroom
stubby
thin
filopodia
(d) P30
Control
2.5% Sevo
2.5% Sevo + Dex
(e) P30
Average dendritic spine density (spines/μm)
ns
ns
2.5
2.0
1.5
1.0
0.5
0.0
Control
2.5% Sevo
2.5% Sevo + Dex
(f) P30
% of dendritic spine types
100
80
60
40
20
0
Control
2.5% Sevo
2.5% Sevo + Dex
*
*
mushroom
stubby
thin
filopodia

Figure S4

Figure S4. Morphological analysis of dendritic spines in adolescent mice exposed to Sevo during embryonic stages. GFP+ pyramidal neurones in the somatosensory cortex at P21 and P30. (a) Representative images of dendrites and spines at P21. (b) Dendritic spine density statistics for control (n = 3 mice; 45 dendrites, 6670 spines), 2.5% Sevo (n = 4 mice; 48 dendrites, 6481 spines), and 2.5% Sevo + Dex (n = 4 mice; 67 dendrites, 8627 spines) groups at P21. (c) Proportion of spine types at P21. (d) Representative images of dendritic structures at P30 for control (4 mice; 39 dendrites, 5699 spines), 2.5% Sevo (4 mice; 40 dendrites, 5421 spines), and 2.5% Sevo + Dex (5 mice; 45 dendrites, 6299 spines). (e) Dendritic spine density statistics at P30. (f) Proportion of spine types at P30. The proportion of filopodia is significantly reduced in the 2.5% Sevo group compared to control and reversed by Dex. Error bars represent SEM. Kruskal-Wallis test, post-hoc: Two-stage linear step-up procedure of Benjamini, Krieger and Yekutieli. *: $p < 0.05$, ns: not significant.
